# Game Theory-Based Authentication Framework to Secure Internet of Vehicles with Blockchain

**DOI:** 10.3390/s22145119

**Published:** 2022-07-07

**Authors:** Manik Gupta, Rakesh Kumar, Shashi Shekhar, Bhisham Sharma, Ram Bahadur Patel, Shaily Jain, Imed Ben Dhaou, Celestine Iwendi

**Affiliations:** 1 Chitkara University School of Engineering and Technology, Chitkara University, Himachal Pradesh 174103, India; manik.gupta@chitkarauniversity.edu.in (M.G.); shaily.jain@chitkarauniversity.edu.in (S.J.); 2 Department of Computer Engineering and Applications, GLA University, Mathura 281406, India; rakesh.kumar@gla.ac.in (R.K.); shashi.shekhar@gla.ac.in (S.S.); 3 Department of Computer Science and Engineering, Chandigarh College of Engineering and Technology, Chandigarh 160019, India; drpatelrb@gmail.com; 4 Department of Computer Science, Hekma School of Engineering, Computing, and Informatics, Dar Al-Hekma University, Jeddah 22246-4872, Saudi Arabia; 5 Department of Computing, University of Turku, 20500 Turku, Finland; 6 Higher Institute of Computer Sciences and Mathematics, Department of Technology, University of Monastir, Monastir 5000, Tunisia; 7 School of Creative Technologies, University of Bolton, Bolton BL3 5AB, UK; celestine.iwendi@ieee.org

**Keywords:** Internet of Vehicles, security, authentication, game theory, physical unclonable functions, duel game, stochastic duel game, blockchain

## Abstract

The Internet of Vehicles (IoV) is a new paradigm for vehicular networks. Using diverse access methods, IoV enables vehicles to connect with their surroundings. However, without data security, IoV settings might be hazardous. Because of the IoV’s openness and self-organization, they are prone to malevolent attack. To overcome this problem, this paper proposes a revolutionary blockchain-enabled game theory-based authentication mechanism for securing IoVs. Here, a three layer multi-trusted authorization solution is provided in which authentication of vehicles can be performed from initial entry to movement into different trusted authorities’ areas without any delay by the use of Physical Unclonable Functions (PUFs) in the beginning and later through duel gaming, and a dynamic Proof-of-Work (dPoW) consensus mechanism. Formal and informal security analyses justify the framework’s credibility in more depth with mathematical proofs. A rigorous comparative study demonstrates that the suggested framework achieves greater security and functionality characteristics and provides lower transaction and computation overhead than many of the available solutions so far. However, these solutions never considered the prime concerns of physical cloning and side-channel attacks. However, the framework in this paper is capable of handling them along with all the other security attacks the previous work can handle. Finally, the suggested framework has been subjected to a blockchain implementation to demonstrate its efficacy with duel gaming to achieve authentication in addition to its capability of using lower burdened blockchain at the physical layer, which current blockchain-based authentication models for IoVs do not support.

## 1. Introduction

As a solitary application of the Internet of Things (IoT), IoV, with the growth in smart cities, is gaining a lot of traction in the fields of research and business. The IoV may greatly increase transportation efficiency, cut energy consumption, and minimize traffic accidents as part of a smart city [1]. Dedicated Short-Range Communication (DSRC) is a wireless communication technology designed for the vehicle environment. DSRC communication uses two types of devices: an on-board unit (OBU) used by the vehicle to communicate with other vehicles (V2V) and a Roadside Unit (RSU) that facilitates communication between the vehicle and everything (V2X) [2]. DSRC is designed to allow for the secure exchange of safety messages between vehicles using the Elliptic Curve Digital Signature Algorithm (ECDSA) [3]. DSRC is believed to be the first ever technology for the establishment of IoV [4]. There are four types of Vehicle to Everything (V2X) communication in IoVs: Vehicle to Vehicle (V2V), Vehicle to Roadside (V2R), Vehicle to Human (V2H), and Vehicle to Sensor and infrastructure (V2S) [5,6] used with dynamic mobile networks to create connections across heterogeneous networks. Intelligent transportation systems will be built on the backbone of V2X connectivity [7,8]. VANETs, as we know them, are evolving into the Internet of Vehicles. While VANETs are made up of simple ad hoc connections between vehicles sharing data, IoV encompasses a larger network that includes people, objects, and other heterogeneous networks. Mobile networks such as long-term-evaluation, fifth-generation technology are included in IoV to offer a more extensive and stable communication network than VANETs currently provide. Communication between IoV and the backbone network is performed using ad hoc networks, which are used to communicate with the infrastructure. It is possible to gather and share data on the surroundings, cars, and road conditions using IoV [9,10,11,12,13].

There are severe concerns about security in the IoV, which impacts the activities of its consumers. IoV security is compromised if an intrusion attack occurs, which may lead to traffic accidents if attackers have access to automobiles. Several occurrences in the past show just how severe the security risks of a connected car may be when they happen. Information that must be transmitted quickly is common in IoV security services. The right to privacy of the sender is violated if any personal information about the sender is leaked from a transmitted communication that has not been protected and encrypted. IoV devices must meet the four most important security standards listed below:

***Anonymity*:** It is important to maintain the privacy of the sender by excluding any information that might identify the sender. There should be no indication that a person or organization is behind a message by its content. An IoV system must protect the identity of the sender who sends a message.

***Data Integrity*:** The sender’s data must be received by the recipient in the same form in which it was delivered. To make sure that no one changed the message while it was being sent, the system should be able to pick up on any attempts to do so.

***Authenticity*:** It is possible for a communication to come from a legitimate source as well as a malicious one. The first step in strengthening the security of the IoV system is to determine whether or not the node is legitimate. Malicious nodes should be found by the system and dealt with. The system should be able to tell the difference between good and bad nodes.

***Low Overhead*:** The majority of IoV communications are time sensitive. If they do not arrive on time, they are of no use to the recipient. So, the IoV system must make sure that, while focusing on security, overhead costs do not go up so much that broadcasting takes so much longer that its no longer useful once the message has reached its destination.

Keeping track of vehicles in a network demands that they interact with each other using their unique identifiers, and in certain situations, the network communications may include confidential information. Attackers have an easier time modifying or intercepting network traffic when sensitive information is made publicly available. Driver and passenger confidentiality are therefore seriously compromised. There was another option that relied on the identification of each vehicle as a signature to cope with this problem: a private key generator that creates a private key for each vehicle based on its unique identity. A major trust issue often arises in this case. However, in a circumstance where a large number of vehicles are involved, none of these scenarios could overcome the constraints. As the number of cars on the road grows, so does the number of certificates issued and revoked. As a result of this, it is critical to identify an attacker who broke the paradigm. Because of this, it became obvious to researchers that they needed to work on privacy management techniques that protect privacy while simultaneously tracing the attacker’s identity.

In the Internet of Vehicles, the trusted authorities’ services are dependent on the vehicle’s uploading of relevant data, and the vehicle’s choices are dependent on the trusted authorities’ services. As a result, the possibility of leaked information or tampering is considerably raised since the vehicles and RSUs communicate over wireless channels. For example, if inaccurate information is used by the trusted authorities to deliver services to automobiles, it might result in financial losses and possibly put vehicle safety at risk. Consequently, the solution to these issues lies in creating an IoV authentication system that is both safe as well as dependable [14].

### 1.1. Problem Statement

Assuming all vehicles can only authenticate with the trusted authorities, the RSU functions as an intermediary node to facilitate communication between the trusted authorities and each individual vehicle, rather than as a primary authentication node. Because of the high pace of vehicle movement, the trusted authorities must perform mutual authentication with all the vehicles that seek authentication in a timely manner. If the number of cars seeking mutual authentication at the same time is considerable, it may be impossible to perform mutual authentication with all vehicles within a given time frame for such centralized authentication models. Consequently, such centralized authentication techniques are susceptible to trusted authorities’ communication and processing resource limitations. Many current authentication schemes rely on just one trusted authority in the network architecture due to the assumption that the trusted authority (TA) has unrestricted access to resources. It is obvious that each region in a smart city should have one or more trusted authorities of this kind in charge of overseeing various departments. As a result, IoVs should use a multi-trusted authority network paradigm. Its long-distance mobility, however, poses an authentication challenge due to the vehicle’s vulnerability to cross-trusted authority spam [15].

### 1.2. Motivation

The decentralized nature of blockchain technology lends itself well to distributed consensus applications [16], so it is appropriate for resolving the IoV cross-trusted authority’s authentication issues. A distributed ledger that maintains vehicle-specific data is made available to all trusted authorities through blockchain technology. The information on vehicles that is kept in the ledger is safe from tampering because of the security afforded by the blockchain technology. In order to make authentication less reliant on a single trusted authority or vehicle, as much of the computation as possible has been moved to the RSU servers.

**Figure 1:**
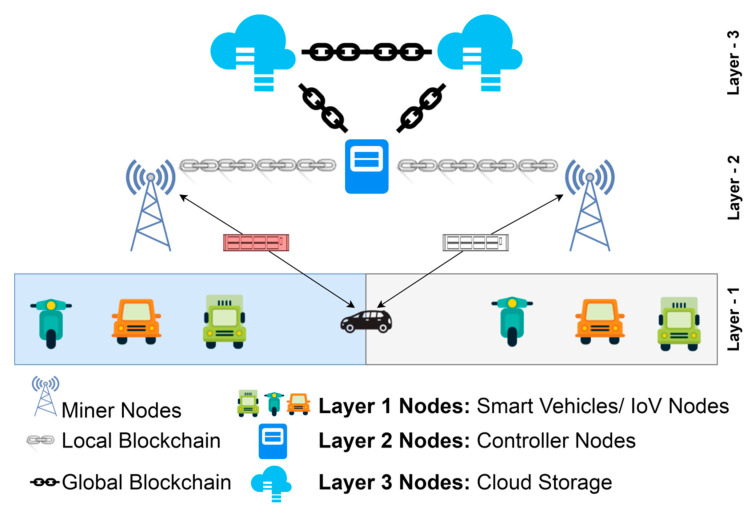
Three-layer blockchain-enabled and game theory-based authentication setup for IoVs.

To address the aforementioned issues, we suggested a three-layer blockchain-enabled and game theory-based authentication mechanism for IoVs in this paper, as shown in Figure 1. Here, all the vehicles representing end nodes of communication are immunized by PUFs [17] against any kind of physical attack and are categorized as layer 1 device. Maintaining the blockchain among high velocity nodes is really a challenge. Hence, all the layer 1 nodes need to communicate through RSUs in a centralized way. These RSUs, along with other controller nodes, communicate through the local blockchain in layer 2. The controller nodes may be either certain special RSUs that have higher computing capabilities than regular RSU or they may be dedicated high-end traffic control stations. These controller nodes further communicate with cloud storage rather than centralized data centers with global blockchain, and both may act as trusted authorities in the network. The following are prime characteristics of the proposed framework:

1. For better authentication efficiency and to reduce communication time, we have proposed blockchain-enabled RSUs that aid authorization by shifting the majority of the burden to the RSUs.
2. Our method uses only a few cryptographic techniques, such as hash operations, XOR operations, and “pseudo-random” numbers, to reduce the amount of time the authentication framework needs to spend computing.
3. The multi-trusted authorities network model that has been proposed in our framework is more realistic. Blockchain technology allows all trusted authorities in our framework to use the same ledger to record vehicle information, which not only enables the cross-trusted authority’s authentication but also improves its efficiency.
4. The proposed framework proposes layer 1 vehicles powered with PUFs to provide lower level authentication via challenge response pair gaming technique, as well as layer 2 and layer 3 authentications via the duel game technique [18]. Hence, providing end-to-end authentication.

### 1.3. Organization of Paper

Section 2 of this paper is a review of the literature on vehicle authentication, which is primarily focused on IoVs but also covers related contributions in VANETs. Section 3 proposes the framework for the authentication of IoVs, followed by a structural setup that divides the framework into three layers. In Section 4, the security investigations are performed. That first covers the basic assumptions of the framework, followed by the formal analysis performed mathematically and an informal analysis that focuses on the possible attacks. Section 5 justifies the implementation of the proposed framework that is further analyzed, evaluated and compared with the related studies in Section 6. Finally, the whole contribution is concluded along with possible future aspects in Section 7.

## 2. Literature Review

The first part of this section analyzes the views and opinions of a number of authors in the field of mutual authentication in the network. The core VANET’s authentication issues are also taken into consideration, along with those of IoVs. Furthermore, Table 1 presents the papers reviewed and compared based on the technologies and techniques they follow.

Bagga et al. [19] developed an IoV-based blockchain-enabled batch authentication technique so that smart cities can use AI-powered smart vehicles. It uses dynamic clusters in which each vehicle broadcasts a message to other vehicles and the road RSU that needs to be verified. The cluster vehicles are authorized by their RSU concurrently in both V2V single authentication and batch authentication methods. A method for RSU to establish a group key that can be used by all of the cluster’s vehicles is also included in these operations. In order to make the suggested model more successful, the authors of [19] implemented a blockchain method employing fog servers and cloud computing. To mine the blocks of transactions, a consensus technique based on practical byzantine fault tolerance voting is used, and big data analytics using AI/ML algorithms were also made possible by the legitimacy and veracity of the massive amounts of data stored in blockchain.

Zisang et al. [20] concentrated on the design of the authentication and key agreement protocols between the vehicles and the trusted authorities. The blockchain produced by all trusted authorities during the registration phase and its connection with the data centers are assumed to employ current technologies, which may represent some idealized assumptions. Using a multi-TA network topology and blockchain technology, the authors of [20] build an RSU-assisted authentication and key agreement protocol for IoV. When compared to other protocols that employ centralized authentication, the authors of [20] use RSU to help with the mutual authentication among the vehicles and the trusted authorities, which may alleviate some of the trusted authority’s authentication efficiency issues related to compute and communication constraints. The trusted authorities in this protocol also create a blockchain network, which solves cross trusted authority authentication of vehicles as well as improves authentication efficiency among them.

Gupta et al. [21] propose a lightweight, safe framework for IoVs that provides robust authentication and communication security based on blockchain. To ensure that low-powered devices may benefit from the security features of the blockchain, it first introduces the concept of a branched blockchain, which takes into consideration the most recently used block. The current mining technologies are not constructed to satisfy the IoV’s requirements at the physical layer. Decentralization, scalability, availability, and load balancing for the physical layer and higher are among the proposed system’s most important features. It is only necessary to keep track of active blocks in the network, rather than complete blocks, in order to further enhance the framework’s lightweight property. The network is able to run on low-powered devices thanks to the creation of lightweight blockchains. Additional features include a distributed hash function and a robust design. Increasing the number of vehicles may be possible because of the robust structure of the vehicle’s framework. Internal tables may be updated when new devices are added and the network’s rate of failure changes, so there is no need for parameter adjusting. In order to show the framework’s lightweight, XOR operations, hash functions, and branching the blockchain may all be employed.

An authentication mechanism should also provide anonymity and untraceability, in addition to security. An IoV-enabled Intelligent Transportation System (ITS) may benefit from a novel mutual authentication and key agreement methodology proposed in [22]. To address the issues during secure communication between entities in an IoV environment, they suggested a novel mutual authentication and session key formation mechanism in an IoV-enabled ITS system. It comprises a mutual authentication technique for secure communication between the selected cluster head and an RSU, as well as a mutual authentication for safe communication between two adjacent vehicles. Aside from facilitating secure communication, it also reduces computing costs by transferring fewer messages.

In the IoV, Radio-Frequency Identification Technology (RFID) is a vital technology that may be used for a wide range of applications, including autonomous toll collection, intelligent parking, and data transmission. To further strengthen the IoV network’s security, unique blockchain-based security architecture for RFID-enabled IoV has been suggested by [23]. Due to the limited resources of RFID devices, privacy and security are key concerns, and the IoV is a time-sensitive network where security is paramount. From a security standpoint, Elliptic Curve Cryptography (ECC), a kind of Elliptic Curve Cryptography, is taken into account. As a result, based on a cryptographic solution, the authors of [23] provide a secure ECC-enabled RFID mutual authentication protocol for IoV. Setting up, tag authentication, and server authentication are the three steps of the proposed protocol. The proposed protocol’s security is evaluated by taking into account the study of security needs and security threats. Security needs such as mutual authentication, availability, and anonymity are all met by the proposed protocol, and various attacks such as Denial-of-Service (DoS) attacks, replays, and cloning attacks are all avoided by the protocol.

The authors of [24] devise a simple IoV-based mutual authentication scheme using cryptographic processes. Clients and servers may generate a secret key using this protocol, which can then be used to securely interact while utilizing the least amount of computing power feasible. In order to meet the requirements of dynamic entities, the lightweight attribute must be guaranteed. As compared to the existing one, the suggested protocol is less expensive and faster to implement.

In [25], Mahmood et al. offer an efficient, conditional privacy preservation domain based on a mutual authentication approach for V2V and V2I communication in a VANET. In this system, each domain is divided into a number of sub-domains, where each sub-domain holds the Certificate Revocation List (CRL) for all RSUs placed inside the domain. For mutual authentication to be successful, the vehicle must verify its identity with the TA. The vehicle may begin transmitting data to other VANET components as soon as the RSU has provided it with a set of pseudo-identities and secret keys. The performance assessment demonstrates that our approach has a lower system cost in terms of computing and communication than other current techniques since it does not employ bilinear pairing. To prevent false communications from being sent out in the likeness of actual vehicles, the authors implement mutual authentication between TA and automobiles. When it comes to VANET security and privacy, they are certain that their plan can meet all of those needs. Finally, the suggested system is better suited to networks of a larger size.

Signatures that are based on a group or pseudonym have issues, including the necessity for certificate distribution and revocation lists. The vehicle must store a valid certificate produced by the management center in such schemes. Prior to message authentication, the receiver must simultaneously verify the CRLs. CRLs need a significant investment in terms of time, space, and computing power. Furthermore, many of these methods are predicated on a TA and do not fulfill real-world requirements. As a result, Jie et al. [26] present an efficient authentication method for VANETs based on semi-trusted authority. Using the self-healing key distribution mechanism with a certificate less signature in a semi-trusted authority environment, the receivers do not need to query the CRLs in this scheme. To avoid wasting storage and communication resources, CRLs are not required to be kept on board the vehicles. As a result, the computing burden is lessened, and the message authentication process operates more quickly and effectively. Additionally, the suggested method is more useful because it comes from a source that can be somewhat trusted. Bayat et al. [27] suggest a method with an RSU-based scheme in which the TA master key is incorporated into a tamper-proof device given at the RSUs. Because of a secure and high-speed communication connection between the TA and RSUs, our method is more feasible than schemes that store the master key on-board devices. The authors of [27] claims that their approach to secure authentication on VANETs is novel, but it was accomplished in this work.

Many papers have presented several identity-based privacy-preserving authentication techniques in recent years to solve security and privacy issues. Libing et al. [28] discovered, however, that none of these techniques are secure enough to safeguard the privacy of their users and are open to intrusions due to their inherent vulnerability or excessive computational complexity. As a result, they concentrate on improving privacy protection via authentication while also improving speed. The flaws of the earlier method are first described in this publication. In addition, an efficient privacy-preserving mutual authentication system for safe V2V communication in VANETs to improve privacy protection and speed is also offered. The authors of [28] explicitly established that in comparison to the prior system, their method can achieve security objectives in dynamic topographical scenarios by security analysis and comparison.

Security and privacy are the most demanding challenges of RFID systems, owing to the rising use of RFID technology in many areas such as health care and finance. Many authentication mechanisms have been proposed to improve the security of these systems. An ECC-based RFID authentication system was presented by Dinarvand et al. [29]. Using authentication methods to tackle these issues is a flexible and effective solution. Hash functions and symmetric cryptography are the foundations of many RFID authentication schemes. The usage of Elliptic Curve Cryptography has expanded because of its short key size, fast calculations, and good security. To tackle the drawbacks of current authentication methods, the authors of [29] present an RFID authentication protocol that uses ECC for mutual authentication. This protocol is found secure enough to avoid various attacks on RFID systems while still meeting the security standards of the RFID authentication protocol.

It is possible for automobiles on the road to create their own self-organizing network and send messages to one another through the VANET. An authentication technique is necessary, however, since data are sent across an unsafe network. Ying et al. [30] have suggested an authentication mechanism for secure vehicle networks, claiming that it could withstand a variety of assaults. In the end, however, Chien et al. [31] found that the protocol proposed by [30] was vulnerable to an offline identity guessing attack, including location spoofing and replay attacks, as well as proved time-consuming for authentication. In [31], an updated approach to address these flaws is suggested.

In the event that the RSU compromises the genuine identity, the suggested technique employs a pseudonym throughout the joining procedure. All prior identity-based methods were vulnerable to insider attacks and failed to withstand revocation. Murtadha et al. [32] proposed a solution that addresses these issues since the vehicle signs the beacon using a signature retrieved from the RSU, which eliminates the need for the vehicle to manually sign the beacon. The prerequisites for message integrity and authentication, privacy protection, non-repudiation, traceability, and revocation are all met by this approach. It also offers conditional anonymity, which ensures that an honest vehicle’s true identity is protected until nefarious activity is discovered. Modification, replay, impersonation, and man-in-the-middle (MITM) attacks are all immune to this system. Despite the fact that many current systems employ bilinear pairing operations; this approach does not work because of the complicated processes that result in considerable processing cost. As a result, there are considerable storage and transmission costs owing to the lack of a revocation list.

## 3. Proposed Authentication Framework for IoVs

**Figure 2:**
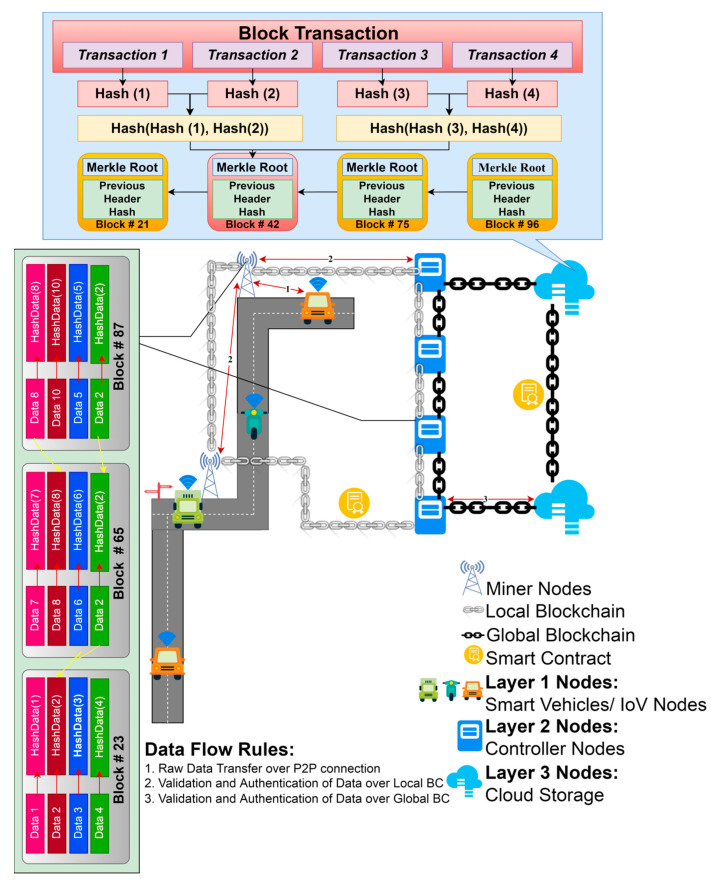
Relational Structure among Network Components.

IoV-networked devices may now benefit from a blockchain-based infrastructure that facilitates layered authentication and authorization. It is recommended that the whole IoV network be divided into three levels by the proposed multi-layer framework. Layer 1 is comprised of IoV nodes that interact in a hybrid mode, i.e., centralized communication among vehicles and RSUs acting as cluster heads of the regional cluster containing vehicles in a particular geography before vehicle authorization and decentralized communication using branched blockchain [21] after successful authorization of the vehicle. Controller nodes and RSUs, which also act as miner nodes and cluster heads of lower layer nodes, respectively, are found in layer 2. The controller nodes and cloud storage are both included in Layer 3. It should be noted that RSUs participate in centralized vehicle communication. However, they interact with other RSUs and controller nodes on the local blockchain; thus, they participate as both layer 1 and layer 2 nodes. Using a lightweight consensus, layer 2 nodes may interact safely in a blockchain system. This layer implements the local permissioned hyperledger fabric blockchain. Similarly, controller nodes serve as a link between cloud storage connected via global blockchain and layer 2 nodes connected via local blockchain, allowing them to participate as both layer 2 and layer 3 nodes. The high-level layer’s advanced security measures and the global blockchain’s implementation provide the highest levels of security and anonymity for all users. All the controller nodes, cluster heads, and vehicle nodes are presumed to have a 5G cellular connection. In order to execute the decentralized blockchain method at Layers 2 and 3, both RSUs and controller nodes have sufficient processing capacity with adequate servers and CPUs, along with the pre-existing high infrastructure at their disposal via cloud storage. Figure 2 depicts the relational structure among nodes in the proposed framework.

### 3.1. Layer 1

Vehicles, nodes, and pedestrians are all included in this layer, along with network components for communications, protocols, and processes for the IoV layer. Based on geographic network capabilities, each cluster is subdivided geographically and associated with RSUs designated as cluster heads. Prior to vehicle authorization, layer 1 vehicles communicate with RSUs serving as the cluster head in a centralized manner, but after authorization, they switch to a decentralized manner utilizing branched blockchains [21].

**Figure 3:**
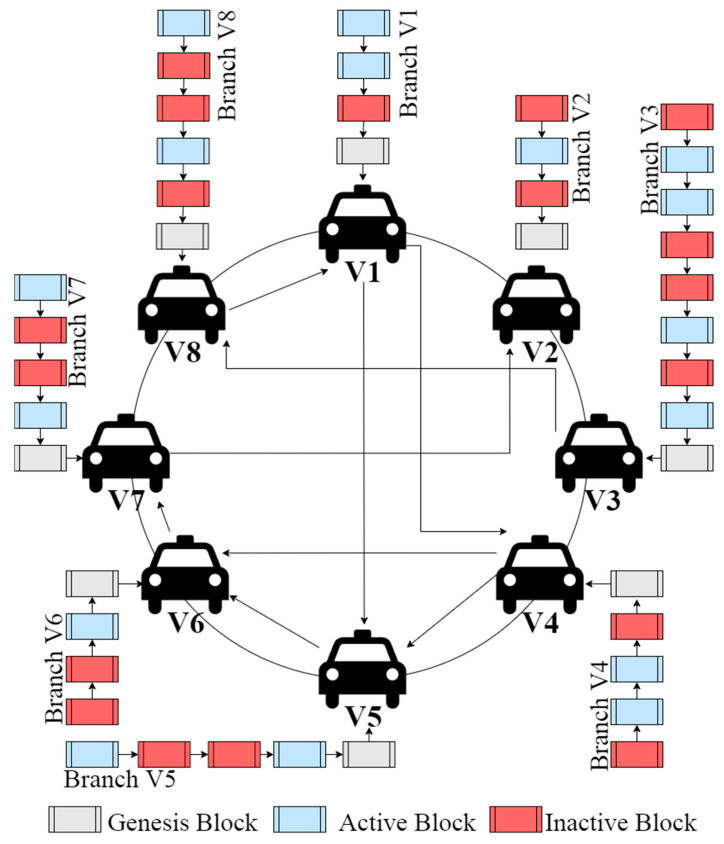
Branched Blockchain for Layer 1 Nodes.

Branched blockchain that seamlessly connects with an existing peer-to-peer network is built on the Chord protocol [33] and Distributed Hash Table (DHT). For the IoV network, it is a data format based on blockchain that makes it possible to securely exchange real-time data while also lightening up the current blockchain by reducing unnecessary overhead from low powered mobile devices. Branched blockchains are remarkable for the fact that they only accept blocks from their own branch, as shown in Figure 3, represented by V1 to V8 in Figure 3. As a result, no one branch’s blocks are in synchronization with all of the vehicles. In addition to creating the genesis block, cluster heads may merge branches into a unified blockchain. The branched block system allows vehicles to distinguish between the active blocks and inactive blocks. Transaction records are stored in these blocks, which are secure ledgers of only active blocks. The hardware requirements for IoV devices differ tremendously because of the wide variety of vehicles that might be included, ranging from low-powered to high-powered devices. A lack of processing memory resources in certain IoVs prevents them from using memory-hard hash algorithms. One of the advantages of branching is that it reduces the amount of space needed to keep all active and inactive blocks by storing just the most recently used block and considering it the only legitimate one in the branch. Because all devices have a single usable block, there is no requirement for memory-hard hash algorithms to keep the mining process from becoming competitive.

**Figure 4:**
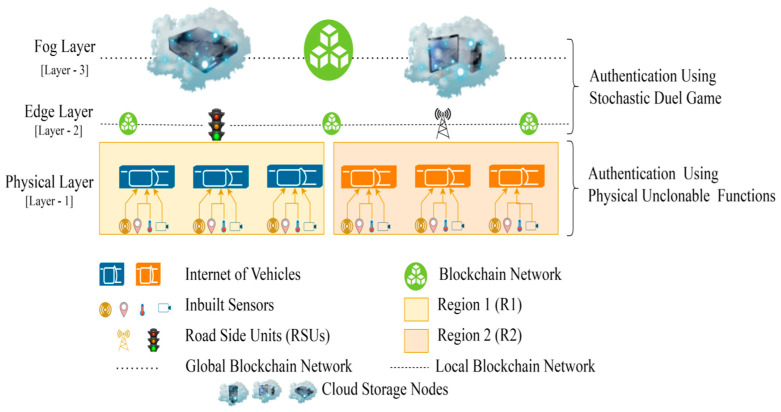
Layer wise Authentication Mechanisms.

**Figure 5:**
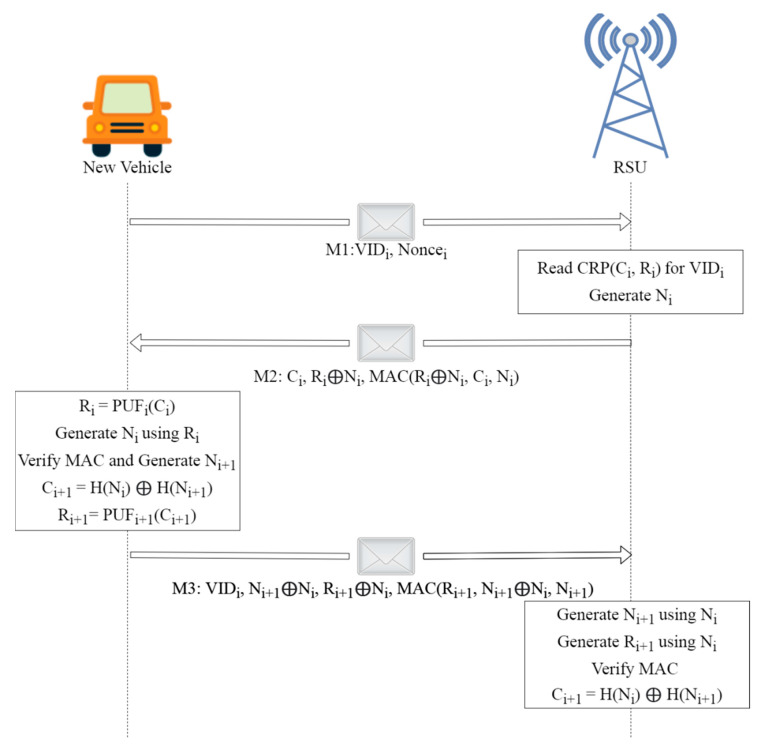
Authentication Using Physical Unclonable Functions.

In our network model, vehicles are assumed to be first outfitted with PUFs, which are then linked over the Internet to an RSU serving as the cluster head in a regional cluster, as illustrated in Figure 4. Challenge–response functions based on the input and physical microstructure of the device may be implemented using PUFs, which are unique in their capacity to detect Integrated Circuits (ICs) [34]. In IoV networks, PUFs may be an effective and low-cost security solution because of their unique properties. When utilized in IoV systems, PUFs may be used to offer security without storing any secrets on the devices themselves. Furthermore, because of the inherent incompatibility of IC microstructure due to manufacturing variances, IoV system components using PUFs are singular at the node level. We presume that the system is setup offline before the execution of this framework by giving an initial CRP to the RSUs. As shown in Figure 5, the authentication procedure of PUF is broken down into the following three messages:

***M1*:** Entering a vehicle is performed by sending the vehicle’s identification number (*VID_i_*) and a random nonce number (*Nonce_i_*) to the RSU.

***M2*:** After locating the *VID_i_* in its memory, the RSU obtains the PUF for this vehicle’s CRP, i.e., (*C_i_*, *R_i_*). Authentication requests are denied, if *VID_i_* cannot be located in the device’s memory. Message 2 is then encrypted using a secret random number (*N_i_*) generated by the server, if *VID_i_* is discovered. In order to ensure the message’s integrity, the server additionally inserts a message authentication code (*MAC*) using the secret random number.

***M3*:** The vehicle then generates a response, *R_i_* from the challenge, *C_i_* using its PUF. Further, using *R_i_*, it obtains a secret random integer and validates the message’s freshness and integrity using the *MAC* that was sent back. Using *N_i_* and *N_i_*_+1_, the vehicle then produces a whole new challenge, *C_i_*_+1_, to solve during the next iteration. For the new secret response *R_i_*_+1_, vehicles must enter a new challenge into the device’s PUF. Message 3 shows that *N_i_*_+1_ and *R_i_*_+1_ are sent safely to the server by the use of *N_i_*. In message 3, the vehicle adds a *MAC* using the *N_i_*_+1_ secret.

The server uses its secret *N_i_* to compute *N_i_*_+1_ and *R_i_*_+1_ and the *MAC* to verify the message. CRP (*C_i_*_+1_, *R_i_*_+1_) against *VID_i_* is then saved in memory by using *N_i_* and *N_i+1_* to generate the new challenge. Once the RSU verifies the *MAC* address of *M3*, it has successfully finished the authentication process. It is impossible to authenticate a vehicle if its *MAC* address fails any of these tests. It is worth noting that *MACs* do not rely on any vehicle-stored secret keys. For authentication, they employ the secret random numbers *N_i_* and *N_i_*_+1_ and to complete the authentication procedure, both the vehicle and the RSU erase all of their temporary variables, such as the values *N_i_* and *N_i_*_+1_ as well as *Nonce_i_* and *R_i_*. The secret random numbers *N_i_* and *N_i_*_+1_ may also be used to construct a secret shared key, e.g., that can be used to encrypt additional communication between the vehicle and RSU.

The authentication process presented here also addresses the security attacks during the authentication processes very well. Assume an adversarial vehicle is attempting to defraud the network by impersonating each new vehicle that joins it. This might be accomplished in general procedures by intercepting the new vehicles produced by *VID_i_* and *Nonce_i_*. The proposed approach, however, denies authentication requests if the device’s memory does not include the relevant vehicle’s *VID*. In the event of an adversary, this scenario is inevitable since PUFs have been proven to be very beneficial for usage as a unique identification for each individual IC. In order to do this, the IC has circuitry that turns the little differences into an exacting digital pattern of 0 s and 1 s, that can be reproduced again and which is similar to a real biometric fingerprint. This pattern is called a “silicon fingerprint”.

To safeguard their data, IP, and operations, vehicles connected to the IoV need keys that are generally embedded by the chip vendors at the early stage of manufacturing. It is more expensive and more difficult to manufacture chips with secret keys since they need to be injected into them by a reputable firm. A PUF or an internal Random Number Generator (RNG) may generate the keys without introducing additional complexity external to the device.

It is not only coming up with a key that is a problem, of course. This is due to the fact that keeping keys in a vehicle securely is not a simple task. Due to Non-Volatile Memory’s (NVM) vulnerability to hardware attacks, secret keys cannot be directly stored in NVM. Increasingly prevalent hardware vulnerabilities enable attackers to view NVM material, making unprotected key storage impractical. This necessitates the development of an alternate method of storing secured keys. Adding a security component to the vehicle is one option. Adding hardware, on the other hand, entails more complexity and expense. The cryptographic keys may be safely stored in silicon PUF without the need for extra hardware.

The following use cases for a PUF in vehicles justify the precautionary measures against early stage attacks on traditional networks at the M1 or M2 level:

**Key Vault:** The creation and storage of a vehicle’s cryptographic root key are the most well-known use case for PUF technology. Key injection is not required in order for the PUF to generate the cryptographic root key, and it cannot be replicated from one vehicle to the other. Due to the fact that a vehicle’s silicon fingerprint serves as its unique identifier, it is never kept. This fingerprint is unique to each chip, making it impossible for an attacker to steal a key from one vehicle in the network and use it to access another.

**Firmware IP Protection:** It is always possible that an IoV device has sensitive information that needs to remain secure. This may be valuable intellectual property that includes trade secrets, or measuring data that is sensitive to privacy or vital to a system. At that point, the vehicles need a secure key vault. Any kind of data may be safely kept in a vault as long as it is tethered to the vehicle’s hardware. Encrypting sensitive data using a PUF’s root key is a simple way to do this.

**Edge-to-Cloud Security:** The vehicle and cloud exchange certificates to create a secure channel based on a public key infrastructure between a vehicle and the cloud, such as a transport layer security (TLS) connection with a cloud service. These certificates serve as a means of establishing trust between two parties. A public/private key pair may be generated from the PUF fingerprint in order to authenticate a device and create an authentication certificate.

Each vehicle is authenticated and approved to ensure the network’s security and privacy using PUF, allowing it to connect with other vehicles in the cluster further via lightweight branched blockchain. RSUs acting as cluster heads then provide lightweight session keys to registered nodes in order to preserve their authorization as an authorization entity and authenticate them to the network. To address scaling issues and the limitations of low-power devices with limited resources, symmetric keys and lightweight cryptography are recommended. The four primary functions of RSUs are to register a new node on the network as a new entity, distribute and assign session keys, manage and initiate connections, and finally create secure communications. The lightweight session keys, also known as the distribution keys, are then encrypted via symmetric key wrapping. A session key is used to safeguard each and every communication. The session key is a symmetric key with a unique identifier and a validity period. Secure communication manages the usage of cryptographic keys for encryption, message authentication, and decryption.

### 3.2. Layer 2

The second tier links all of the controller nodes and RSUs, which serve as cluster heads, collecting and forwarding data to the upper tier. To obtain a consensus based on the stated consensus method, all of the nodes in the second tier execute a private local blockchain. For each cluster, blocks are broadcast to the controller nodes and RSUs, which perform block generation and verification, as well as handle communications between non-consensus devices and nodes within the same tier. This layer is served by the hyperledger fabric (HLF) blockchain platform. While addressing the IoV network’s resource constraints and dispersed vehicle movement, the suggested network architecture must take safe cluster head communications into account. It takes a lot of computing power and time to mine a blockchain. Because of this, it is not appropriate for use on physical layer vehicles. As a result, a distributed consensus-based, lightweight, private, and decentralized blockchain-based data transmission mechanism is proposed. The resource constraints of physical layer vehicles must be taken into account, and an efficient cryptography system must thus be constructed.

Transactions are carried out by peers on a distributed ledger. A peer node may be an endorser and a committer at the same time, or it can perform both of those things. The network’s orderers are responsible for placing all orders. In addition, orderers suggest additional blocks and try to get support for them. The ordering service is a group of nodes that place orders. By default, every other peer is a committer. When an order is placed, the service transmits a block of transactions to the committers, who then maintain the ledger. When a new block of transactions is validated by a peer, the peer stores the changes locally as a copy of the ledger and applies them to the blockchain. Peers may also serve as endorsers for transactions, as long as they have the authority to do so. After the smart contract is executed by the endorser (ChainCode in HLF), it is signed by the endorser and sent back to the client with its cryptographic signature, known as an endorsement. The hyperledger network’s authentication services are provided by the Membership Service Provider (MSP) along with the duel game authentication services.

All the cryptographic techniques and protocols involved in issuing certificates, verifying certificates, and authenticating users are abstracted away by an MSP. The MSP must authenticate the identification of the network nodes. The hyperledger framework is represented logically by the organizations. They are in charge of overseeing the network’s members with the assistance of MSP. Private or dedicated channels are used to link the network’s various components, allowing them to communicate with one another. It is the responsibility of the committers to verify and update the shared ledger. Hyperledger blockchain is built using data transactions for data collection and transfer. Smart contracts are used to specify the terms of transactions. Various peer-to-peer connections may use the ordering cluster to manage transactions and queue orders. Transaction blocks are created by the ordering service and broadcast along with the messages. Many factors, such as the network topology, influence whether vehicles in a blockchain network are designated as endorsers or committers.

To add blocks to the blockchain, the committer node performs validation tasks and updates the network’s state. Once a request for endorsement has been submitted, the vehicle is officially recognized. For approval and consistency monitoring, the endorser node receives this request and forwards it to this node. The execution of the smart contract continues the process of verifying that the smart contract’s code is correct. The endorser responds to the connected vehicle requests by sending back a response and granting particular access for reading and writing. Ordering clusters in the ordering service generate transaction blocks. All CH nodes get the transaction blocks. This level of the blockchain system maintains the ledger and adds transactions and vehicle specifications to it.

### 3.3. Layer 3

In this layer, the controller nodes serve as the administrators of certain RSUs. In a similar way to a cloud server, controller nodes manage devices, create data, and respond to queries in a similar way. The trustworthy nodes in this layer have significant computing capabilities, but with fewer power and processing limits. As a result, the global blockchain is being used to propose stronger asymmetric cryptography algorithms at this layer. Autonomous mining jobs are carried out by the controller nodes at this layer, which do not need access to central authentication servers. Moreover, additional benefits of authentication are provided at this layer by duel game principles. In this layer, the nodes are capable of doing computations in a dispersed network structure. Because of this, it is possible to implement a globally compatible blockchain such as Ethereum coupled with more advanced security measures. For this layer, asymmetric encryption such as Elliptic Curve Cryptography (ECC) is an acceptable option.

Data integrity is ensured by using a blockchain-based system, which increases privacy and security. There is no central node in the upper levels, and the devices are data-independent. The transactions between the nodes at this tier are recorded in the blockchain network. Among the other network members, the trust relationship service mechanism is started by the RSUs, controller nodes, and computing edge nodes throughout the network. The peer-to-peer nature of the blockchain is ideal for a globally dispersed security system. Blockchain-based communication with certificates is used to facilitate communication between controller nodes and computational edge nodes. The distribution of certificates in the blockchain system is maintained by the smart contracts, which allow for a secure connection between various nodes in this layer. The certificates must be signed by the controller nodes. By using a blockchain-based approach, the two controller nodes and linked cloud storage can work together to authorize respective entities with more confidence. While each controller node establishes contact with other nodes in independent clusters, the trust level is raised as a result of this interconnectivity.

It is possible to eliminate the need for domain names and fixed addresses by leveraging the blockchain-based system’s smart contract execution. In the proposed framework, the RSUs do not require fixed addresses or domain names to communicate with the edge devices and execute smart contracts.

While updating the local as well as the global blockchain, consideration is given to anomalies that may develop with any adversary, as its computational capability is very fast with the duel game model. To do this, each node contributing to the blockchain networks, either local or global, must validate its neighbors, which maintains a list of those neighbors. The initial point of communication must be calculated for each node pair. In order to create communication between two nodes, the first step is to determine which node is at a higher risk of being targeted by a threat, and the second step is to determine whether that node is targeted by any threat at the same moment or not. If this occurs, the likelihood function of a node to avoid threat with the node under consideration is set to high, and the scanning node will be added to the set of neighbors, updating the success probability matrix to include the new node and the set of nodes based on the sorted order of communication establishment. A low chance of threat avoidance is established with the neighbor set and the nodes based on the sorted order of communication, which means it will not be regarded as the node for communication, and therefore there will not be any change in the likelihood matrix of successful communication. As a result of these facts, the neighbor list interacts for the shortest amount of time possible in order to construct a smart contract among them. In addition, they enable the data flow between the nodes engaging in a blockchain that may be global or local.

Consider the following scenario: a vehicle moves from one region to another, as shown in Figure 1 and Figure 4. Each of the framework’s vehicles replicates its data over three distinct blockchain networks that are accessible at three separate layers, namely branched blockchain at layer 1, hyperledger fabric at layer 2, and global blockchain at layer 3. Once it joins the networks during the authentication procedure, the vehicle only has to authorize itself once. However, each vehicle will be evaluated as legitimate or not legitimate throughout each iteration cycle of communication depending on the likelihood that it may pose a danger to the communication.

Each node is examined for authorization during the first phase of authorization and authentication based on the CRP of the PUF model. During this time, the node only connects with nearby RSUs or controller nodes, so the vehicle must wait until the first authorization. In our framework, we have separated the territories into regions, where each region may function as a separate city or as a distinct section of a single city. The vehicles can stay connected to the framework using the global blockchain at layer 3, so if they move within other regions as well, authorization will not be needed again. All old vehicles in the new region can be updated through the zone’s cluster head so that the zone’s cluster head can add the new vehicle to the branched blockchain in the following iteration. Technically, the vehicle is always linked to the network, but it may only join the branched blockchain of a new region during the next iteration cycle in which its probability of threat needs to be calculated again. The duration of this iteration cycle is determined by the number of vehicles in the network.

## 4. Security Investigations

This section is divided into two parts: formal and informal security analyses, with some basic assumptions discussed beforehand. Table 2 contains the list of acronyms required here for the security analysis.

### 4.1. Assumptions

To invesitigate the framework from security perspective, certain assumptions have been taken into account and are listed as below:

1. Every vehicle/node is equipped with a PUF.
2. The data from the IoV is sent through a public network.
3. There are two categories of adversary: an internal attacker who has been granted access to data but whose illicit activity is difficult to detect; and an external attacker whose threats are only somewhat less severe.
4. The adversary cannot penetrate data transferred through the private channel in the IoVs, which is one of two types of communication—public and private communications. The malicious node, on the other hand, has access to data delivered through a public channel and may alter, delete, or retransmit it.

### 4.2. Formal Security Analysis

**Lemma** **1.** *Physical attacks cannot be imposed by any adversary on any vehicle/node in the network*.

**Proof.**  PUFs are unique to each vehicle. A miner delivers a challenge C_v_ to the vehicle while the authentication step is in progress. The vehicle will then make use of this challenge in order to create the secret response R_v_. Consequently, R_v_ is never stored in memory and is only activated when required by the vehicle. As a result, an attacker who conducts a physical attack on the vehicle is unable to acquire the secret response R_v_, even if the vehicle survives the attack. This proves that an opponent cannot extract or disclose a vehicle’s secret answer. □

**Lemma** **2.** *Data over the blockchain is immutable, in general*.

**Proof.**  The decentralized nature of a blockchain implies that its network is scattered across several computers called nodes. This removes the possibility of a single point of failure. Each transaction is referred to as a “block”, and the chain of transactions is referred to as a “blockchain”. A block has cryptographic elements that distinguish it. The hashing algorithm of a network dictates the specifics. A transaction becomes rigid by making it difficult to reverse the hashed value. Each block in a chain includes a subset of the preceding block’s contents. Due to the fact that the hash will have a different output when it is re-engineered by an adversary, the resulting block will be out of synchronization with other blocks and hence rejected by the system. If the block being re-hashed is in the middle of the chain, an attacker would have to re-hash earlier blocks to align their historical stamp with the current block. This significantly increases the difficulty of hacking the blockchain [35].

The practice of adding transaction information to digital ledgers that are maintained by blockchain technology is referred to as mining. Mining entails creating the hash for the transaction block, which guarantees the security of the blockchain. When an adversary in the network gains control of a blockchain’s mining capabilities, this is known as a “51 percent attack”. It means that the attackers will have more than half the mining power and will be able to mine more quickly than everyone else. Such attacks are exceedingly difficult, if not impossible, to carry out on a large blockchain network in general. □

**Theorem** **1.** *A node can create a communication channel with any other node in the network by checking the high threat avoidance time*.

**Proof.**  Let *A_n_(t)* be the payoff function of each node *n* in the network, where *n* ranges from 1 to *N* at time *t_n_*. For a node *n*, the pay-off function can be represented by a repetitive non-decreasing function as in Equation (1):

(1) $$0\;\leq \;A_{n}(t)\;\leq \;A_{n}(t+∆)\;where,\;\{\begin{array}{c} t\epsilon \;\left[0,\;t_{n}^{max}\right)\\ \Delta \;\geq 0\\ t_{n}^{max}\epsilon {\mathbb{R}}_{+} \end{array}$$

The probability function that it may come with a threat message can be represented by Equation (2):

(2) $$P_{n\;}^{T}\left(t\right)=\frac{A_{n}\left(t\right)\;}{A_{n}\left(t_{n}^{max}\right)}\;where,n\epsilon \;\left\{1,\;2,\;3,\ldots ,N\right\}$$

Additionally, the probability function of a node to avoid threat can thus be represented by Equation (3):

(3) $$P_{n\;}^{A}\left(t\right)=1-P_{n\;}^{T}\left(t\right)\;where,n\epsilon \;\left\{1,\;2,\;3,\ldots ,N\right\}$$

The *one-to-n* duel game model advises the node communication to select the moment when the probability of avoiding the threat is maximum. Let m and n be two nodes in the network, and they are trying to interact at a time p without knowing each other’s computing ability. Let p be the high time when node *n* maximizes the chance of avoiding threats in the continuous time domain *t*, represented by $t_{n}^{p}\;$by Equation (4):

(4) $$t_{n}^{p}=argmin\{\begin{array}{c} P_{0}^{A}\left(p\right)-\overset{N}{\underset{i=1}{\prod }}(1-P_{i}^{A}\left(p)\right)\geq 0\\ p\geq 0 \end{array}$$

The probability of secure communication establishment for all nodes in the network, represented by Equation (5), is an arbitrary continuous incremental function that reaches 1 when $t_{n}^{p}$ meets the maximum allowed time, p*_max_,* i.e., $t_{n}^{p_{max}}$*,*$t_{ń}^{p_{max}}$< **∞**.

(5) $$P_{n\;}^{A}\left(t\right)=1;\;\mathrm{when}\;t_{n}^{p}=t_{n}^{p_{max}}$$

Let $t_{\left(n,\;ń\right)}^{p_{1}}$ be the first point of time when a set of nodes in the network establishes successful communication. Applying the basis of duel game concepts, it can be represented by Equation (6):

(6) $$t_{\left(n,\;ń\right)}^{p_{1}}=argmin\{\begin{array}{c} P_{n}^{A}\left(p\right)-\overset{N}{\underset{i=1}{\prod }}(1-P_{ń}^{A}\left(p)\right)\geq 0\\ ń=-nandn\epsilon ń\epsilon \;\left\{1,\;2,\;3,\;\ldots ,\;N\right\} \end{array}$$

So, if a node *m* wishes to communicate with a node *n*, it will first check the high threat avoidance time to decide on the establishment of the communication channel and can be represented as in Equation (7):

(7) $$t_{\left(m,n\right)}^{p}=argmin\{\begin{array}{c} P_{m}^{A}\left(p\right)-\overset{N}{\underset{i=1}{\prod }}(1-P_{i}^{A}\left(p)\right)\geq 0\\ t_{\left(m,n\right)}\geq 0 \end{array}$$

It should be noted that any node can create a communication channel with any other node in the network at certain periods of time, which is why the success probability of communication establishment with threat avoidance is a continuous function, but the time series becomes discrete. □

**Theorem** **2.** *The possibility of establishing a communication link between any pair of nodes is independent from other nodes in the network*.

**Proof.**  Each node in the network will have at least three iterations and a maximum of *N* − 1 iterations, where *N* is the total number of nodes in the network and a set of nodes in the network tries to establish the communication link based on the rules of duel-game accepting the presence of some adversary nodes in the network as well. Considering probability theory ideas, the probability triplet (Ω, F, *P*) for time space $T^{n}$ (representing node *n*) is a mathematical construct that offers a formal description of a random process consisting of three components:

**Ω:** the collection of all potential outcomes, i.e., a sample space.

**F:** a space in which events occur, i.e., event space is a set of outcomes in the sample space.

***P*:** a probabilistic function that assigns a probability to each event in the event space, with probabilities ranging from 0 to 1.

From Equation (4), the optimal threshold of the player *n* can be determined into one value in the time domain of a duel game. Let, the node *m* on the *i*-th iteration at $t_{\left(m,n\right)}^{i}$ has the highest probability of threat avoidance in comparison to the probability of threat of other nodes. The instance when any two nodes say *m* and *n* in the network should wait is $t_{\left(m,n\right)}^{i}$, the node *m* at *i*-th iteration cannot achieve highest probability of establishing communication with node *n* until node m reaches the time space $T_{ex_{i}^{m}}^{m}$ and n reaches $T_{ex_{i}^{n}}^{n}$ then the time space event, $T^{n}$ can be represented as in Equation (8). Here:

(8) $$T^{n}\;≜\;\sum_{x}^{}T_{i}^{n}\{\begin{array}{c} n\epsilon \;\left\{1,\;2,\;3,\ldots ,N\right\}\\ T_{i}^{0}=0\;and\;T_{i}^{0}<\\ x\geq 0 \end{array}T_{i}^{1}<T_{i}^{2}<\cdots <T_{i}^{N}$$

This time space event occurs in the probability space $F_{T^{n}}\;$which is also a renewal point process with the following notation of the nodes, as represented in Equation (9).

(9) $$\tau_{i}^{n}≜\{\begin{array}{c} 0\;\;\;\;\;\;\;\;\;\;\;\;\;\;,\;\;i\leq \;0\\ T_{i}^{n}-T_{i-1}^{n},\;\;i>0 \end{array}$$

and the exit index may be represented as in Equation (10).

(10) $$ex_{i}^{n}≜\sum_{x}^{N}\left(\begin{array}{c} N\\ x \end{array}\right)\tau_{i}^{n}\{\begin{array}{c} x\geq 0\\ n\;\epsilon \;\left\{1,\;2,\;3,\ldots ,N\right\} \end{array}$$

Hence, communication during the *i*-th iteration may last till MIN$\left(T_{ex_{i}^{m}}^{m},T_{ex_{i}^{n}}^{n}\right),\;\mathrm{where},\;m=-ń$. The model aims at a restricted duel game process for node *n* in the network while considering the trace algebra, σ, when the node *n* in the network calculates the best time of communication establishment with the majority of the nodes in the network and it can be represented by Equation (11).

$$\sigma =F\;(Ω)\;\cap \;\left\{P_{n}^{A}\left(T_{ex_{i}^{n}}^{n}\right)+P_{ń}^{A}\left(T_{ex_{i}^{ń}}^{ń}\right)\right\}\cap \;\left\{T_{ex_{i}^{n}}^{n}\leq T_{ex_{i}^{ń}}^{ń}\right\};\;\mathrm{where}\;P_{n}^{A}\left(T_{ex_{i}^{n}}^{n}\right)+P_{ń}^{A}\left(T_{ex_{i}^{ń}}^{ń}\right)\geq 1$$

Since there are *N* number of nodes in the network, the possibility of establishing a communication link by any node with another node is independent from other nodes in the network because of backward induction inspired by the duel game process [18,36,37,38]. Thus, determining threat avoidance time is high when a node passes the threshold and is more analytical regardless of their trust value [36]. □

**Theorem** **3.** *The order of threat avoidance by the node pairs to establish communication links in between can be determined*.

**Proof.**  The trust value is directly proportional to the response which other nodes in the network get while calculating the threat avoidance of the corresponding node. The best time for threat avoidance is calculated from Equation (7) and the sorted list of nodes obtained at the initial stage from Equation (6) is as in Equation (11).

(11) $$T_{0}^{A}=argsort_{\left(m_{i},\;n_{i}\right)\;}\left\{m_{i},n_{i}{}_{i}\;|\;p_{0\left(m_{1},\;n_{1}\right)}^{A}\leq \cdots ..\leq p_{i\left(m_{i},\;n_{i}\right)}^{A}\leq \cdots ..\leq p_{i_{0}\left(m_{i_{0}},\;n_{i_{0}}\right)}^{A}\right\}$$

(12) $$\forall p_{0}^{A}=\left\{p_{0\left(m_{1},n_{1}\right)}^{A}\leq \cdots ..\leq p_{i\left(m_{i},\;n_{i}\right)}^{A}\leq \cdots ..\leq p_{i_{0}\left(m_{i_{0}},\;n_{i_{0}}\right)}^{A}\right\};wh\mathrm{ere},\;i_{0}\;=\left(\begin{array}{c} N\\ 2 \end{array}\right)$$

Here, in Equation (12), $n_{i}$ is the node evaluating other nodes in the *i*-th iteration. The order of evaluation of one specific node *n* in the network when the total number of sets $T_{n_{0}}^{A}$ and $p_{n_{0}}^{A}$ are *N* − 1, can be relatively defined as in Equations (13) and (14).

(13) Tn0A=argsort(n, ń) {∃〈n, ń〉iń | p(n, ń)ińA;where ińϵ {1, 2, 3, …, N−1}}

(14) pn0A=argsortp(n, ń){∃p(n, ń)ińA;where ń=−n and ińϵ {1, 2, 3, …, N−1}}

Now, based on the hostile duel game principle, which is actually meant for multiple players and multiple nodes in our case, let us consider the change in the status of pair-wise communication between any two nodes $m_{i}$ and $n_{i}$ in terms of sorted order of establishing communication among the n-nodes in the network during the *i*-th iteration, this set of nodes can be interpreted by Equation (15):

(15) $$T_{i_{0}}^{A}=\left\{{\left\{m_{1},n_{1}\right\}}_{1},\ldots \;,\;{\left\{m_{i},n_{i}\right\}}_{i},\;\ldots ,{\left\{m_{i_{0}},n_{i_{0}}\right\}}_{i_{0}}\right\}\;;where\;i_{0}\;=\left(\begin{array}{c} N\\ 2 \end{array}\right)$$

Far from the *1-to-n* duel game principle [38] that may allow any adversary to get a single attempt of attack on every other node in the network and makes duel game concept more complicated, the proposed hostile duel game principle changes the status of every node after every attempt to make the duel game principle more relevant with actual network scenarios where any adversary may attempt to threat the network infinitely, thus, the status of every node is changed after every attempt for communication and the success probability of all the nodes need to be calculated after every iteration. If any node is listed as a non-trusted node, then the success probability of the particular node is changed to zero. Let, $P_{\sigma }^{C}$(t,$N_{i}$) be the success probability matrix for pair-wise communication establishment in the *i-th* iteration and can be defined by Equation (16).

(16) $$P_{\sigma }^{C}\left(t,\;N_{i}\right)=\left\{P_{1\;}^{A}\left(t\right),\;P_{2\;}^{A}\left(t\right),\ldots \ldots ,\;P_{N-1\;}^{A}\left(t\right),P_{N\;}^{A}\left(t\right),\right\}$$

When node *n* establishes communication with node ń, then the success probability matrix $P_{\sigma }^{C}\left(t,\;N_{i+1}\right)$ and set of nodes based on the sorted order of communication establishment, $T_{i_{1}}^{A}$ is updated as in Equations (17) and (18), respectively.

(17) $$P_{\sigma }^{C}\left(t,N_{i+1}\right)=\left\{\begin{array}{cl} P_{\sigma }^{C}\left(t,N_{i}\right) & |\mathrm{Communication}\;\mathrm{failed}\;\mathrm{to}\;\mathrm{establish}\\ P_{\sigma }^{C}\left(t,N_{i}\backslash \{\hat{n}\}\right) & |\mathrm{Communication}\;\mathrm{successfully}\;\mathrm{established} \end{array}\right.$$

(18) $$T_{i_{1}}^{A}=\left\{\begin{array}{cl} T_{i_{0}}^{A} & |\mathrm{Communication}\;\mathrm{failed}\;\mathrm{to}\;\mathrm{establish}\\ T_{i_{0}}^{A}\backslash \left\{\{n,ń\};\;ń\;\epsilon \left\{\begin{array}{c} 1,2,3,\ldots ,N\\ \mathrm{and}ń\neq n \end{array}\right\}\right. & |\mathrm{Communication}\;\mathrm{successfully}\;\mathrm{established} \end{array}\right.$$

Since the order of threat avoidance by the nodes can be ascertained by backward induction, each set of nodes in the current iteration and the best time to establish a communication link in between. It can be defined by Equations (13) and (14).

Every node has *N* − 1 iterations during high threat avoidance time and can communicate with other trusted nodes in the network. Looking back on Equation (14), the set of order of communication establishment with other nodes by node *n* can be established as follows:

(19) $$p_{n_{i}}^{A}=\left\{p_{i\left(n,1\right)}^{A}\leq p_{i\left(n,2\right)}^{A}\leq \ldots \leq p_{i\left(n,N-1\right)}^{A}\right\}$$

□

**Corollary** **1.** *For a node in communication, it is possible to find the optimum iteration cycle to avoid threats and establish blockchain security*.

**Proof.**  Considering the two-person duel game concept, the probability of threat avoidance by node *n* is analogous to the complement of the probability of threat by other nodes, ń in the network [39] and can be applied to the pair-wise duel game in *i*-th iteration. So, the success probability of node *n* to establish communication with node ń during the *i*-th iteration can be defined in Equation (20) and the high threat avoidance indicator in Equation (21), respectively, as below:

(20) $${Ƥ}_{i\left(n,ń\right)}^{C}=\{\begin{array}{c} 1\;\;\;\;\;\;\;\;\;\;\;\;\;\;\;\;\;\;\;\;\;\;\;\;\;\;\;\;\;\;\;\;\;\;\;\;\;\;\;\;\;\;\;\;\;\;\;\;\;\;\;\;\;\;\;\;\;\;\;\;\;\;\;;where\;\;\;n=ń\\ 1-P_{ń}^{A}\left(P_{i\left(n,ń\right)}^{A}\right)\overset{N-1}{\underset{i=1}{\prod }}\left\{P_{i}^{A}\left(p_{i\left(n,ń\right)}^{A}\right)\right\};\mathrm{where}\;n\neq ń \end{array}$$

(21) αi(n,ń)A=Ƥi(n,ń)CƤi(ń, n)C∀ń=−n;where i=1, 2, 3, ….,(N2)

Deriving from Equations (19) and (21), the optimum iteration cycle with high threat avoidance time with most compatible node, ${ń}_{i_{n*}}$ that satisfies the blockchain security during iteration $i_{n}^{*}\;$can be defined as follows:

(22) $$i_{n}^{*}=argmin_{i}\left\{i\;|\;\alpha_{i\left(n,ń\right)}^{A_{1}}\leq \ldots \leq \alpha_{i\left(n,ń\right)}^{A_{x}}\right\};\;\forall ń=-nandp_{n*}^{A}=p_{\left(n,{\;ń}_{i_{n*}}\right)}^{i_{n*}}$$

where x→∞, and is the total number of attempts to avoid threat for secure communication establishment.

Considering Equation (22), we can derive from Theorem 1, Theorem 2, and Theorem 3 that security threats can be avoided by finding the optimum iteration cycle and establishing secure blockchain communication. □

**Corollary** **2.** *For a node pair in communication, mutual authentication is established*.

**Proof** **.** From Theorem 1, it is clear that by verifying the high threat avoidance time, a node may construct a communication channel with any other node in the network. The sequence in which node pairs avoid threats in order to establish communication links between them can be identified by Theorem 3. Hence, it is clear that before exchanging or retrieving information, all entities involved in the message exchange scenario verify each other to ensure their legitimacy, thus forming mutual authentication between them. □

### 4.3. Informal Security Analysis

In this section, we explore security problems to demonstrate that our proposed framework is safe against all important security threats. Some of the major security threats are analyzed here informally in Table 3, which contains the propositions along with the security defense mechanisms against the known attacks.

## 5. Framework Implementation

Each vehicle node has its own blockchain account and uses the contract to communicate with other nodes. It is worth noting that each RSU node maintains a blockchain snapshot that is synchronized with its peers. Algorithm 1 specifies that the simulation process begins with the vehicle permission phase, during which the region’s cluster head broadcasts a “hello” message along with the nonce to all new vehicles joining the network. This then proceeds to the phase of session key distribution, when the distribution key is supplied, or when the existing distribution key is updated. When it comes to the first scenario, the vehicle and its intended message are both responsible for providing the distribution key, while the public key of the cluster head is used in the second scenario. The cluster head then passes a collection of legitimate session keys in both cases. However, in the second case, the cluster head also passes a different packet containing the public key of both the cluster head and the vehicle. The session keys are then added to the list of permissioned vehicles for that specific vehicle. Additionally, during the communication initialization stage, if a vehicle is unable to locate the session key using SessionKey ID, it launches a session key request to get one. Otherwise, the message is encrypted with an authentication code to be delivered end to end along with the session key. When the new vehicle is added to the list of permissioned vehicles, the P2P network sets the flag for the communication to true.
**Algorithm 1:** Vehicle Permission**function:***VehPer* **input:**new_vehicle **output:** true/false
**procedure:**  get((‘hello’, nonce_CH), CH) ← v∗[n].node  **if**dist_key==1 **then**   get(vehicle_ID, sessionKeyRequest_ID, nonce_CH, nonce_v, distKey) ← v∗[n].node   get(nonce_v, SessionKey[ ], distKey) ← CH  **else**
  get(vehicle_ID, sessionKeyRequest_ID, nonce_CH, nonce_v, PK(CH)) ← v∗[n].node   get(nonce_v, SessionKey[ ], distKey) ← CH   get(distKey, PK(CH), PK(veh)) ← CH  set(SessionKey[v]=1, CH) ← permissioned[v]  search(commReq_ID, SessionKeyID[v], nonce_v, distKey)  **if**commReq_ID==1 **then**
  get(nonce_v, nonce_s, SessionKey[ ])← permissioned[v]  comm._Flag[v] = true  **else**   comm._Flag[v] = false  return **comm._Flag[v]**


Further, validation of neighbors needs to be performed by Algorithm 2, in which a list of neighbors is set at each node contributing to the blockchain network. Every node pair needs to calculate the first point of communication. If a node *i* wishes to communicate with a node *j*, it will first check the high threat avoidance time to decide on the establishment of the communication channel, and both the nodes will achieve the highest probability of establishing communication until they reach the same time–space, i.e., communication is successfully established between both the nodes. If this happens, then the probability function of a node to avoid threat with the node in consideration is set as high, and the scanned node will be added to the neighbor set and the success probability matrix will be updated with the new node along with the set of nodes based on the sorted order of communication establishment. Otherwise, the probability of threat avoidance is set to low with the neighbor set and the set of nodes based on the sorted order of communication remains the same, i.e., it will not be considered as the node for communication and hence there will be no change in the success probability matrix. Based on these facts, the neighbor list interacts for the minimum time space exit index during which they can establish smart contracts among them, and furthermore, they allow the data flow through Algorithm 3 among the nodes interacting in a blockchain that may be global or local.
**Algorithm 2:** Validate Neighbours for BC Network***function:****safeNodeBC* ***output:****Validated Neighbours* ***procedure:*** ***for****i in*$n_{*}$,$n_{*}\epsilon N$*∀∗=1, …, n **do***   *adj.n∗[i].node={}← create empty sorted list of adjacency nodes*  ***for****j in*$n_{*}$,$n_{*}\epsilon N$*∀∗=1, …, n and j ≠ i**do***    $t_{\left(i,\;j\right)}^{p_{1}}$*← Calculatefirst point of time for communication*   ***if***$t_{\left(i,\;j\right)}^{p}$*==*$t_{\left(i,\;j\right)}^{p_{max}}andT_{ex_{}^{i}}^{i}$*==*$T_{ex_{}^{j}}^{j}$***do***     $P_{\left(i,\;j\right)\;}^{A}\left(t\right)=1$    *adj.n∗[i].node ← j*      $P_{\sigma i}^{C}\left(t,\;N\right)=P_{\sigma i}^{C}\left(t,\;N\left\{j\right\}\right)$      $T_{i,\;j}^{A}=T_{i-1,\;j-1}^{A}\;\backslash \;\left\{\left\{i,j\right\};\;\forall i\neq j\right\}$    ***else***      $P_{\left(i,\;j\right)\;}^{A}\left(t\right)=0$      $P_{\sigma i}^{C}\left(t,\;N\right)=P_{\sigma i}^{C}\left(t,\;N\right)$     $T_{i,\;j}^{A}=T_{i-1,\;j-1}^{A}$   *return **adj.n******∗***

**Algorithm 3**: Data Flow***function:** DataFlow(n_1_, n_2_)* ***output:** updated_BC* ***procedure:*** ***while**simulation**do*** *T, B← initialize* ***for** x ← 1 to T **do***  ***for** y ← 1 to B **do***   *Transfer data from n_1_to n_2_*   *Block ← b(y)*  *Validate Block on n_2_*  *Deploy Block from n_2_to BC*

In Algorithm 4, the method to find the successor operation has been improved to make use of the name tables. No matter how many vehicles are in the chain of succession, it is possible to verify the identity of a newer one with the check successor function. The alternative is to have *v* search for the vehicle *v*_0_ in its name table and thereafter call Algorithm 4 at *v_0_*, whose *ID* is closest to *ID*. We chose *v*_0_ because of its proximity to *ID* and the amount of information it possesses about the area.
**Algorithm 4:** Check Successor***function:****FindSuccessor* ***input:****ID* ***output:****successor* ***procedure:*** ***if** ID∈ (v, successor)**then***  *return **successor*** ***else***  ***for** i = v_o_ to 1**do***   ***if** name[i]∈ (v, ID) **then***    *returnAlgorithm 4← **name[i]***   *returnAlgorithm 4← v*

The server then calls Algorithm 5 and awards a genesis block to thenewly permissioned vehicle when it certifies that it possesses the session key.

Algorithm 6 illustrates the whole simulation of the proposed framework, which comprises a set of RSU nodes designated by R and a set of vehicles designated by *v*, which are both initialized by Algorithm 5.
**Algorithm 5:** Node Initialization***function:****NodeSetUp* ***input:****n* ***procedure:***  *genesis.block← create* *n ← generate node* *n.node← create account* *n.node.account← allocate resource*  *n.predecessor = null* *n.successor = null*

**Algorithm 6:** Simulating the Framework***while****simulation**do*** *//Initialization* ***for****i in r∗, r∗∈R∀∗ = 1, 2, 3, …, n **do***   *Algorithm 5← call*  ***for****j in i**do***   *x_i_∗[j].node← compile contract*   *x_i_∗[j].node← deploy* ***for** i in v∗, v∗∈V∀∗= 1, 2, 3, …, n **do***  *Algorithm 5← call* *//Network Update* **for** i in x*, x*∈(v*, r*) **do**   ***for***
*j in adj.m∗,*
$m_{*}\epsilon N$*∀∗=1, …, n **do***
   *if Algorithm 1(new_v)== true**then***     *Algorithm 5← call*     contract ← sendMessage() ← v*[i].node
    r∗[j].node← contract ← v*[i].node
    ***while***
msgrequest(v*[i].node)==1 **do**
     ***for***
*k in v∗, v∗∈V*
*∀∗=1, 2, 3, …, n **do***
      ***if***
*v∗[k].cert ==*
*true **then***
       *v∗[k].cert← Permission*
       *generate v∗[k].ID*       ***for** m in k**do***
        *v_i_∗[m].node← compile contract*
        *v_i_∗[m].node← deploy*            *v_i_∗[m].predecessor = null*            *v_i_∗[m].node.successor = reg_veh.find_suc(v)*
        *sp = { v_k_∗[m].predecessor, v_k_∗[m].node.successor}*        ***if**sp∈ (v, v_k_∗[m].node.successor)**then***          *sp = v_k_∗[m].node.successor*        ***if** ((v_k_∗[m].predecessor == null)or v_i_∗[j].predecessor*
*∈ (v, v_i_∗[j].predecessor)**then***          *v_k_∗[m].predecessor = reg_veh*
         *name[t++] = Algorithm 4(v + 2^t−1^)*      ***else if**v∗[k].reg ==1 **then***        ***while***msgrequest(v*[k].node)==1 **do**        ***if** PUFCRP(v∗[k]) ==1 **then***
         *v∗[k].cert ← Issue*
         *Set v∗[k].cert = 1*        ***else***
         *v∗[k].cert ← Reject*
         *Set v∗[k].cert = 0*      ***else***
       *v∗[k].cert ← Reject*
       *Set v∗[k].cert = 0*  *//Update LocalBC*  ***for***
*i in*
$r_{*}$,
$r_{*}\epsilon L$*∀∗=1, …, n **do***   *Algorithm 2← call*  ***for** j in adj.m∗,*
$m_{*}\epsilon N$*∀∗=1, …, n **do***
   ***while***
*MIN*$\left(T_{ex_{}^{i}}^{i},T_{ex_{}^{j}}^{j}\right)$***do***     contract ← sendMessage() ← r*[i].node
    r∗[j].node← contract ← r*[i].node   *Algorithm 3(MinerNode, ControllerNode)← call*
  *Clear Block from b_B-1_ to b_B-N_*  *//Update GlobalBC*  ***for***
*i in r∗, r∗∈G∀∗=1, 2, 3, …, n **do***   *Algorithm 2← call*  ***for** j in adj.m∗,*
$m_{*}\epsilon N$*∀∗=1, …, n **do***
   ***while***
*MIN*$\left(T_{ex_{}^{i}}^{i},T_{ex_{}^{j}}^{j}\right)$***do***     contract ← sendMessage() ← r*[i].node
    r∗[j].node← contract ← r*[i].node   *Algorithm 3(ControllerNode, CloudServer)← call*
  *Update GlobalBC*
  *Replicate and filter data from GlobalBC to Cloud_Server*

Then, throughout the lifecycle of the process, whenever the transactions are carried, it depicts the information flow arrangement in which the vehicles communicate with the contract, which in turn connects with the blockchain via RSUs. A vehicle must first attain permission before it may produce data. Additionally, vehicles may be registered and deleted using their blockchain account addresses. They maintain track of all blockchain permissioned vehicles. Additionally, *PUFCRP()* function keeps track of the vehicles’ PUF CRPs. RSU also acts as the certificate authority and keeps track of all certifications given to the vehicles. The enforcer verifies whether a vehicle is permissioned when it produces data. If the vehicle is on the permissioned list, a PUF challenge is issued to it, and if the challenge is accepted, the connection between the car and the local blockchain is successfully formed. The RSUs then provide a certificate to the vehicle, which is used for its authentication, after completing these inspections. As a result, the vehicle is no longer required to do these examinations again after receiving the certificate. The certificates granted to vehicles are valid for as long as they are set with permissions on the local blockchain, which is a one-time process. Vehicles that are no longer permissioned will have their certificates revoked, and they will have to go through the process of getting permission and then a certificate all over again.

Further, in Algorithm 6, the local and global blockchains are updated based on considerations of anomalies that may arise with any kind of adversary, even if their computing capacity is really fast.

## 6. Performance Analysis and Discussion

This section details the basic environmental setup along with the performance evaluation of the proposed framework and discussion of the comparative analysis of computational and transaction overhead with the existing methods.

### 6.1. Initial Setup

In order to show the viability and practicality of the proposed blockchain architecture, we created simulation models in three distinct contexts connected to each level of the multi-layer network. The first part of the Layer 1 implementation involves authorizing and registering new vehicles through the PUF model, and the second involves connecting to a branched blockchain network using Chord. RSUs, controller nodes, and APIs are implemented in Layer 2 of the hyperledger fabric blockchain. Etherum and hyperledger fabric metrics are being compared in the global blockchain deployment simulator at Layer 3.

The first phase of cluster head registration was developed in Java, and the PUF model was implemented in Matlab that allows clients to get authorized through the cluster head and Node.js is further used for running server and client entities. The client and peer, both written in Python, are also supplied. Privileged vehicles in the Chord ring may communicate with each other through the peer network. Each node is aware of its predecessors and successors in the Chord architecture. An *ID* of a vehicle, *VID* is generated for each vehicle as soon as it joins the Chord network. In order to enter the ring, the new nodes must first interact with any active node and choose their successor. To avoid linear search, each node must store a name table with *i* entries, where *i* is the bit length of the hash key. The nth node in the network will reveal a successor to the node x’s entry ((*x + 2n* − 1) *mod c*) that has not yet been discovered. Every node sends a query towards the next antecedent or descendant using the key’s name table, depending on that key’s location within the network. Both the nodes that join the framework and the nodes that fail or depart on their own must be handled by Chord. Nodes’ successor pointers are maintained using a basic stabilization technique that is sufficient to assure the correctness of query execution via lookups. Using the pointers to the successors, the name table entries may be checked and corrected quickly and accurately. If any of the Chord ring’s nodes have been destroyed by connecting, a search that occurs before stabilization has concluded may exhibit one of three behaviors.

Assuming the first scenario is correct, the lookup only takes *O(log n)* steps to discover the correct successor among the name table entries involved. The second scenario has successor pointers that are valid but names that are erroneous. This result in correct lookups, but it may take a little longer. Third, nodes in the affected region may contain erroneous keys or pointers to the successors that have not yet relocated to the newly linked nodes, causing the failure of the search. To begin with, it should be noted that vehicles are assumed to be equipped with PUFs, which are connected to CRPs on the network in Layer 1.A smart contract language, Solidity was used to create the enforcer contract, and Python V.3.7.3 was used to create the dPoW consensus mechanism, to build the IoV-blockchain network architecture.

An integrated programming environment for Solidity called Remix was used to write and construct this smart contract. A small amount of web3.js was used for the RSU and vehicle nodes. Web3.js offers a set of libraries for communicating with an Ethereum node over HTTP, IPC, or WebSocket.

Hyperledger fabric, Docker version 17.06.2-ce, and cURL were used to build up the basic environment for Layer 2. There was also Node.js V8 used in conjunction with this Go Programming Language 1.12 in order to write chaincode applications at this layer. In addition to this, Git Bash was added to provide a more favorable shell environment. When executing the blockchain applications on a workstation, a Layer 3 simulation model was built. The Ethereum and hyperledger private network throughput and latency metrics were easily measured in this setting. The networks were all set up in the same way and given the same amount of virtual workload. An Oracle virtual machine, the VirtualBox client, was used to run the simulation models on an Ubuntu OS workstation (version 17.04).

Specifications for this workstation include the following details: Nvidia GeForce GPU, which has 2754 MB of memory, the Intel Core i5-3210M 2.5 GHz processor with 4 GB of DDR3 RAM. The shell programming environment was set up using Git Bash Terminal. The nodes were created in the terminal using the Ethereum Go client (Geth), which is a command-line interface written in the Go programming language. For transaction delivery, web3.js was used on both the RSU and the vehicle side to interact with their respective Geth clients and host the local blockchain at layer 2, as well as to host a global blockchain across controller nodes and cloud storage on the RSU side.

### 6.2. Framework Evaluation

The RSUs and controller nodes’ energy usage and time overhead are evaluated via Omnet++ simulations. In the branched blockchain, it is the RSUs that take up the most resources; in the local blockchain, it is the controller nodes that use up the most energy since they need to process all transactions and execute many symmetric and asymmetric hashing and encryption operations. The most computationally demanding operation for the vehicles at Layer 1 is symmetric encryption, and most vehicles have the ability to accomplish this work. The suggested framework’s overhead is compared to another technique that does not require encryption, hashing, or a ledger but has the same transaction flow as RSUs and controller nodes, and we call this the baseline method. To meet our simulation’s resource limits, we employ iPv6 as the fundamental communication protocol. The RSUs get data from 15 z1-mote sensors every 20 s, which we mimic for the vehicles. The findings are averaged over five minutes of simulation time. Data are stored on a controller node that is linked directly to RSUs and then sent to the cloud through a controller node. With this approach, we can get a complete picture of how the system works. We simulated two distinct and real-time traffic flow patterns for storing the transaction. In the first scenario, cloud storage is periodically backed up by vehicles. However, in the second scenario, when a vehicle receives a query from another vehicle anywhere in the network, the data are stored in the vehicle. The comprehensive information on the parameters utilized during the simulation is given in Table 4.

Evaluation criteria further include the percentage of attacks that succeed and total packet overhead. The worst-case situation is that a trustable controller node, which has created more than 70 blocks and hence acquired a high degree of trust, generates a new block containing one fraudulent transaction. A successful assault is one in which the forged block is not identified by any of the honest controller nodes or RSUs throughout the course of twenty simulations. When compared to a baseline where the overlay network is constructed such as Ethereum, the packet overhead is lower in the proposed framework.

**Figure 6:**
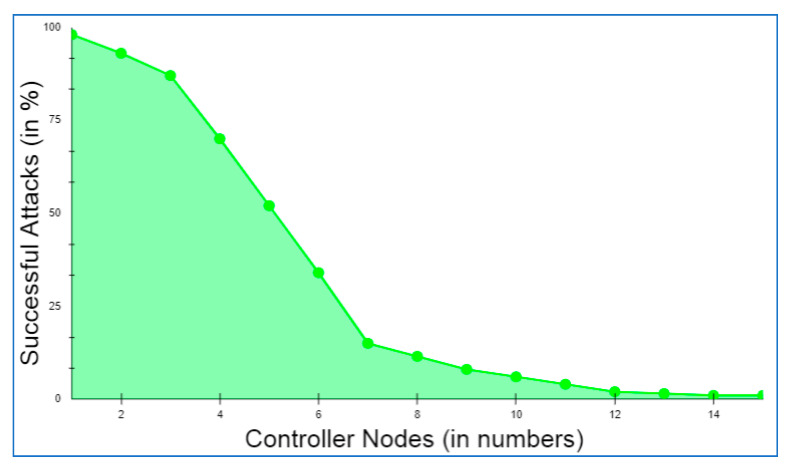
Success percentage in the attacks with respect to the number of controllers.

**Figure 7:**
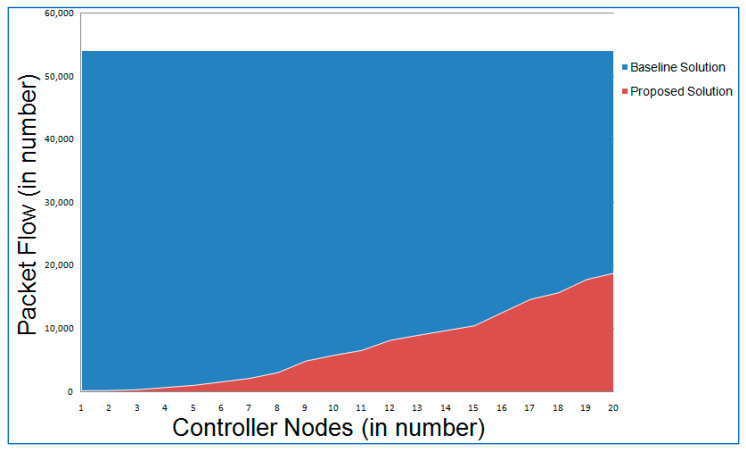
Packet flow comparison with baseline based on number of controller nodes.

Remember that in Bitcoin, all the Layer 2 nodes, 70 in our case, containing both RSUs and controller nodes, control the blockchain, but only a few of them are responsible for managing the blockchain. Because all transactions in a block are authenticated, the baseline would always be able to identify the attack. Figure 6 and Figure 7 depict the findings. A successful attack is far less likely as the number of controller nodes grows. The number of controller nodes has a linear effect on the amount of packet overhead. All attacks are identified with 15 controller nodes. However, the overhead of the relevant packets is much lower than the baseline.

**Figure 8:**
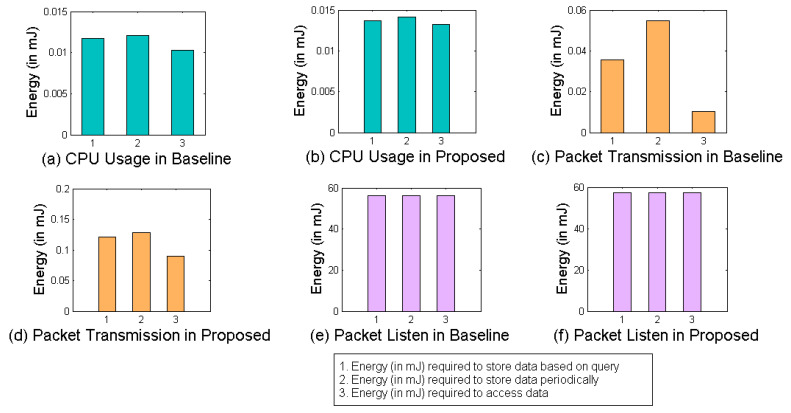
Energy consumption in baseline model and proposed framework at different instances.

The results of the energy usage are shown in Figure 8. The proposed framework primarily uses energy for three primary functions: CPU usage, transmitting packets, and listening packets. As a consequence of encryption and hashing, the proposed framework results in longer packets. This doubles the transmission energy usage. It should be emphasized that in our analyses, we have assumed that the radio is always on. An increase in the relative listening overhead would result if the radio was turned off for short periods of time to save energy.

The proposed framework is simulated on two different wireless MAC protocols, i.e., IEEE 802.11a and IEEE 802.11p, as both the protocols are versatile and recommended for IOVs. IEEE 802.11a provides a 20 MHz channel bandwidth and a data transmission rate of up to 54 Mbps. IEEE 802.11p, on the other hand, offers a 10 MHz channel bandwidth and a maximum data transfer speed of 27 Mbps. In addition, the number of carriers, modulation, and coding rate are the same for both the protocols. Table 5 details the specifications of both the protocols in detail.

**Figure 9:**
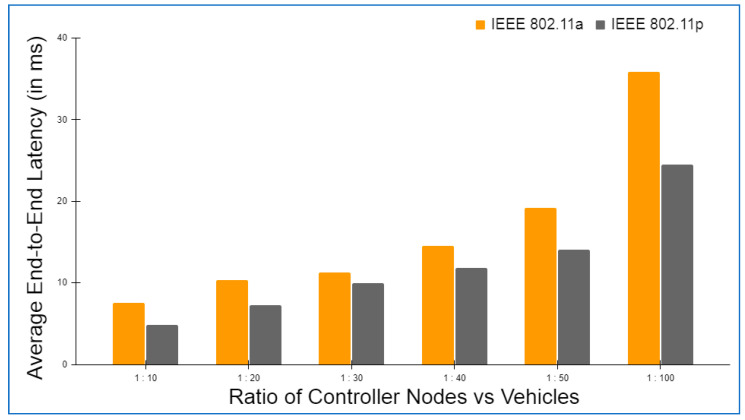
End-to-End Latency Time in the proposed framework for IEEE 802.11a vs. IEEE 802.11p.

**Figure 10:**
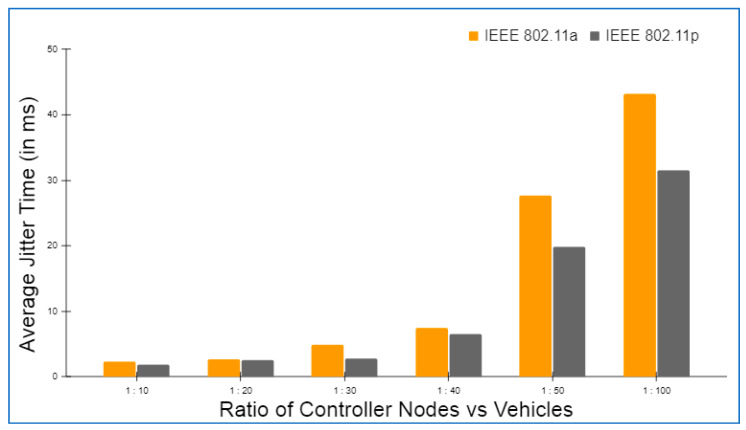
Jitter Time in the proposed framework for IEEE 802.11a vs. IEEE 802.11p.

**Figure 11:**
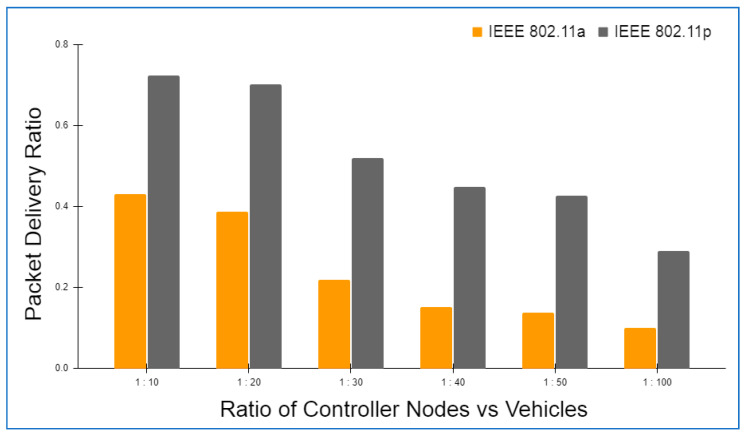
Packet Delivery Ratio in the proposed framework for IEEE 802.11a vs. IEEE 802.11p.

**Figure 12:**
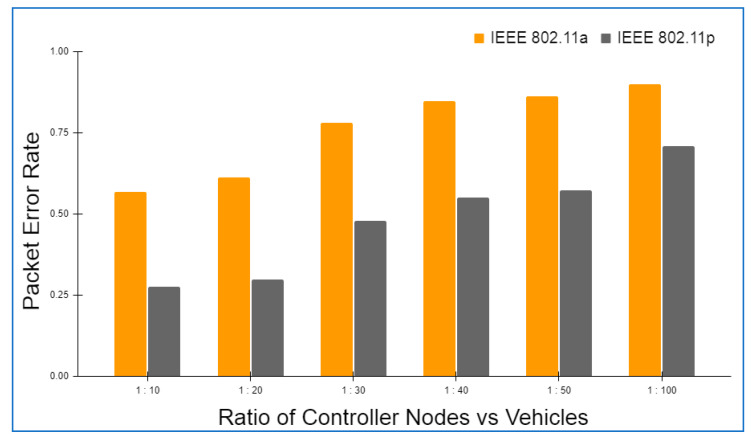
Packet Error Rate in the proposed framework for IEEE 802.11a vs. IEEE 802.11p.

Both IEEE 802.11a and IEEE 802.11p are primarily affected by the number of vehicles participating in the network. The number of controller nodes merely affects the result of any variation. Figure 9, Figure 10, Figure 11 and Figure 12 show the outcomes on parameters such as end-to-end latency, jitter time, packet delivery ratio, and packet error rate, respectively, based on the variation in the number of vehicles in the fixed area of 10 km^2^ on the AODV routing protocol for both the wireless protocols, i.e., IEEE 802.11a and IEEE 802.11p. The complete iPv6 packet (header and payload combined) must fit under the link layer’s maximum transfer unit (MTU), which for an IEEE 802.11 MAC frame is 2312 bytes. The iPv6 payload size has been limited to 2272 bytes with a base header of 40 bytes in order to prevent fragmentation overhead. Since the UDP header is set at 8 bytes, the UDP payload is fixed at 2264 bytes. Six different scenarios are considered in each of the comparisons, wherein the ratio of controller node or RSU to the number of vehicles ranges from 1:10 to 1:100. This assures the branched blockchain network at Layer 1 contains the number of peer vehicles ranging from 10 to 100.

In order to acquire the average end-to-end latency in Figure 9, we computed the arithmetic mean of all the packets’ end-to-end latencies. The network jitter in Figure 10 is the fluctuation in the time it takes for a packet to arrive. The packet delivery ratio in Figure 11 measures the proportion of successfully received packets to all packets transmitted. The packet error rate in Figure 12 measures the proportion of packets that were transmitted in error but were not received compared to the total number of packets that were sent. There are several cross-layer properties that cause packets to be lost during transmission; it could be bit error rate at the physical layer, contention at the MAC layer, or congestion at the transport layer.

### 6.3. Operational Cost

On Ethereum, completing a job requires spending “gas”, which is the declared cost of the activity, such as the completion of a transaction or the execution of a smart contract. *wei* is the smallest unit of ether, the virtual currency of the Ethereum network. In other words, 1 ether is equal to 10^18^ wei. There is a direct correlation between the amount of gas utilized and the cost of a contract. As a result, more gas is required to do a more challenging task. We were able to assess the gas consumption of the proposed framework and its activities using the Remix IDE. Executing the enforcer contract needs 23,126 units of gas, while deploying it requires 10, 12, 132 units of gas. The gas estimate is unrestricted while a message or authorization is being sent to a vehicle. Their input size is not defined owing to the variable length data, which is restricted to 0.5 KB, and the ability to add limitless vehicles.

### 6.4. Comparative Analysis

On the basis of the computation and transaction overhead, this section compares the proposed authentication framework with the IEEE 802.11a wireless MAC protocol as it has higher channel bandwidth and data transmission rate than IEEE802.11p. This framework has been compared to other relevant models that are already in use, such as those developed by Bagga et al. [19], Xu et al. [20], Gupta et al. [21], Bagga et al. [22], Sharma et al. [23], Vasudev et al. [24], Al-Shareeda et al. [25], Cui et al. [26], Wu et al. [28], Dinarvand et al. [29], Chen et al. [31], Bayat et al. [27], and Alazzawi et al. [24]. On the basis of their ability to safeguard against impersonation attacks, offline password guessing attacks, replay attacks, linking attacks, man-in-the-middle attacks, Denial of Service (DOS) attack, 51% attacks, public BC modification attacks and physical attacks, the models in Table 6 are contrasted against all the security propositions that were discussed in Table 3. Table 6 shows that the proposed framework outperforms all the security propositions. The special catch among them is to guard against physical cloning and side-channel attacks that become possible with early authentication at Layer 1 of the PUF model.

#### 6.4.1. Computation Overhead

An IoV node’s lifespan is directly impacted by the framework’s overall computation overhead, and that should be kept to a minimum where authentication steps are involved. First, we need to figure out how long it will take to implement the different procedures in the compared models. Table 7 shows how our framework stacks up against other approaches in terms of computational overhead. For computation overhead, seconds or milliseconds are used as units to measure the amount of time it takes to do a certain task. The symbols *H*, *L*, *n*, *M*, *I*, *A*, *S*, *B*, *SED*, *E*, *X*, *N*, *P*, *O*, *s* and *tt*, respectively, represent hash operation, the length of forward and backward hash chain, number of random integers generated by RSUs, scalar multiplication operation, inversion operation, addition operation, subtraction operation, bilinear pairing operation, symmetric key encryption/decryption, encryption operation, modular exponentiation, number of signature verifications, pairing, map to point, server operations, tag operations.

For overhead calculation, it is necessary to first determine the amount of time it takes to complete different procedures in the compared models. To see how our protocol stacks up against others, Table 7 can be referred to. Vehicles require 8*H* + *log*_2_*vH* + 4*X* + 1*MA*Coperations and also the RSUs/controller nodes require 8*H* + *log*_2_*vH* + 4*X* + 1*MAC* operations for the authentication process for *v* vehicles.

**Figure 13:**
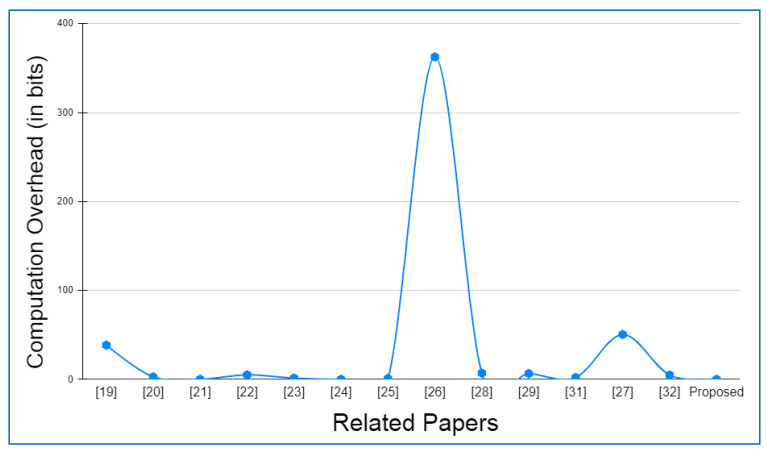
Computation Overhead Relative Comparison.

This proves that the proposed protocol requires very low processing power for authentication, which translates into low energy requirements as well, in comparison to existing authentication schemes. The 0.000296 ms required for the XOR operation is no longer relevant since the hash computation time has been greatly reduced. To offer a secure authentication protocol, XOR (⊕) and MAC operations are considered trivial, and the hash operation is extremely cheap in the application of IoVs that compute a total computational overhead of (16 + log_2_v)H on the proposed framework. Figure 13 and Table 8 show that the proposed framework has a total computation overhead of 0.0337 ms, in which the vehicles, RSUs/controller nodes each one of them significantly contributing 48.12%, i.e., 0.01622 ms for (8 + log2v)H and 0.88% is involved in each of the XOR operations, and further MAC operations involve 0.24% that is considerably less than or almost equivalent to most of the comparison models considered for IoVs. It can be observed that though the framework’s computation overhead is on the lower edge, it still is not able to outperform a few, but the strong defense against physical cloning attacks and sybil attacks makes the slight increase in the overhead justified as the comparative studies do not deal with these attacks.

#### 6.4.2. Transaction Overhead

An implementation of the proposed framework employs a method that takes 0.0021 milliseconds to generate a hash and conduct an XOR operation. Using a workstation with a 2.5 GHz Intel Core i5-3210M CPU and 4 GB of DDR3 RAM, we were able to determine that the framework can process data at a maximum rate of 1.8 MHash/s. In Layer 1, the output of the PUF model is considered as 128 bits and the size of messages and time stamps are each considered as 64 bits. The vehicles generate random nonces of 64 bits and so do the cluster heads, symmetric key encryption and decryption of size 256 bits; session keys of 64 bits and 64-bit symmetric distribution keys. This comes with a total of 1280 bits of communication between vehicles and RSUs.

**Figure 14:**
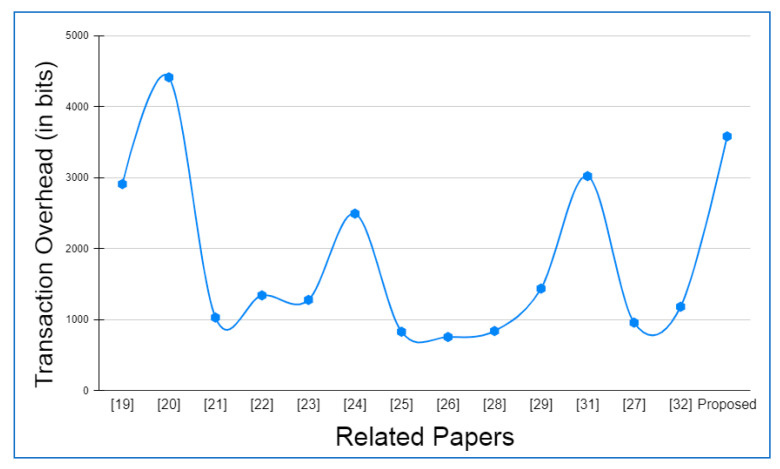
Transaction Overhead Relative Comparison.

As an additional authentication parameter, each auxiliary parameter of authentication has a 256-bit value and is reliant on pointers to relevant tuples, transitory secret parameters, and the master key of 256 bits, along with the time stamp of 64 bits in Layer 2 as well as Layer 3 to which it is linked. This computes a total of 576 bits in each layer. Thus, a total of 2432 bits is measured as the transaction overhead of the framework. This cost may be viewed as higher than a few of the mentions in Table 9 and Figure 14, but it must be noted that though the comparative studies use TA to authenticate vehicles and messages, all of them are unable to tackle the major bottlenecks of physical cloning and side-channel attacks, which are major concerns.

## 7. Conclusions and Future Work

The openness and self-organization of IoV make it a target for malicious attacks. In this paper, blockchain-enabled game theory-based authentication for IoV security is proposed to address this issue. PUFs, duel gaming, and a dPoW consensus process are used to ensure that vehicles may be authenticated from initial entrance through travel into other TA’s areas without delay. The framework’s trustworthiness is further strengthened by formal and informal security studies. A thorough investigation shows that by comparing the proposed framework to other existing competing systems, a thorough investigation shows that it achieves higher levels of security and functionality while also providing better communication and lower computational costs. Though the communication and computation overhead seem a bit higher than a few related studies, the multi-level authentication in this three-layered framework gives it more strength to defend against a number of attacks, especially side-channel and physical cloning attacks, which all the related studies are unable to provide. This paper presents three highlighted contributions: first is the enhanced security with blockchain along with PUFs and duel-gaming based authentication; second is the ability to use lightweight blockchain compatible with the physical layer, which is termed as branched blockchain, further evolved to HLF-oriented local blockchain at layer 2 and Ethereum-based global blockchain at layer 3; and third is the ability to deal with physical cloning and side-channel attacks.

There are a number of ways this work might be extended in the future. Though most studies prefer bitcoin-based blockchain systems [43], the Tron [44] blockchain network may be considered in the future due to its low gas fees and fast transaction rate to reduce communication overhead. The authors of [45] presents several blockchain applications, which motivates future integration of IoVs with blockchains to prevent scams, cargo distributions inspired by [46], and validation of pool-based vehicles or third-party driver services [47]. Furthermore, the proposed duel-game-based mutual authentication technique, in addition to the use of lattice cryptography, can open up a new vertical for dealing with future quantum computing challenges. The IoT combined with the IoV has a wide range of applications and potential applications, but it also has a wide range of difficulties that need to be solved, such as device-network human interfaces, security, and privacy. There is a huge diversity of data, a large amount of data, and a lack of standard design, all of which are problems. One problem is the architecture of this network, which should be scalable; the latency rate should be reduced with high bandwidth [48,49].

## Figures and Tables

**Table 1:** Related Work in Authentication of IoVs and VANETs.

Paper	Year	Network Type		Multilayer Authorization	Blockchain Usage	Authentication Technique
		VANET	IoV			
[19]	2021	N	Y	N	Y	Batch authentication
[20]	2021	N	Y	N	Y	Roadside Unit-assisted authentication
[21]	2021	N	Y	N	Y	Blockchain-based authentication
[22]	2021	N	Y	N	N	Mutual authentication and key agreement
[23]	2021	N	Y	N	Y	Elliptic Curve Cryptography-Enabled Radio Frequency Identification Mutual Authentication
[24]	2020	N	Y	N	N	Cryptography-Based Authentication
[25]	2020	Y	N	N	N	Elliptic Curve Cryptography (ECC) and general one-way hash function
[26]	2019	Y	N	N	N	Authentication based on semi-trusted authority
[27]	2019	Y	N	N	N	RSU-based authentication
[28]	2019	Y	N	N	N	Cryptography-Based Authentication
[29]	2019	Y	N	N	N	Elliptic Curve Cryptography-Enabled Radio Frequency Identification Mutual Authentication
[31]	2019	Y	N	N	N	Cryptography-Based Authentication
[32]	2019	Y	N	Y	N	Cryptography-Based Authentication

Note: Y: Yes; N: No.

**Table 2:** Acronyms Used in This Paper.

Acronym	Definition
***A_n_*** **(*t*)**	payoff function of each node *n* in the network, where *n* ranges from 1 to *N*, at time *t_n_*
$t_{n}^{max}$	maximum allowed time for an iteration cycle
${\mathbb{R}}_{+}$	set of positive real numbers
$P_{n\;}^{T}\left(t\right)$	probability function that it may come with a threat message
$P_{n\;}^{A}\left(t\right)$	probability function of a node to avoid threat
$t_{\left(n,\;ń\right)}^{p_{1}}$	first point of time when a set of nodes in the network establishes successful communication
$t_{\left(m,n\right)}^{i}$	instance when any two nodes say *m* and *n* in the network wait to establish communication
(Ω, F, *P*)	Probability triplet $\mathrm{for}\;\mathrm{time}\;\mathrm{space}\;T^{n}$(representing node *n*)
Ω	collection of all potential outcomes, i.e., a sample space
F	space in which events occur, i.e., event space
*P*	probabilistic function that assigns a probability to each event in the event space
$T^{n}$	time space representing node *n*
p	time when two nodes try to connect
$t_{n}^{p}$	high time of p when node *n* maximizes the chance of avoiding threats in continuous time domain *t*
$ex_{i}^{n}$	exit index of node *n*
PUF	Physical Unclonable Function
IoT	Internet of Things
IoV	Internet of Vehicles
dPoW	Dynamic Proof of Work
V2X	Vehicle to Everything
V2V	Vehicle to Vehicle
V2R	Vehicle to Roadside
V2H	Vehicle to Human
V2S	Vehicle to Sensor
RSU	Roadside Unit
RFID	Radio-Frequency Identification Technology
ECC	Elliptic Curve Cryptography
(DoS)	Denial of Service
CRL	Certificate Revocation List
VANET	Vehicular Ad Hoc Network
MITM	Man-in-the-Middle Attack
IC	Integrated Circuits
MAC	Message Authentication Code
MSP	Membership Service Provider
HLF	Hyperledger Fabric
DHT	Distributed Hash Table

**Table 3:** Informal Security Analysis.

Serial Number	Propositions	Attack Description	Security Defense Explanation
**P1**	The proposed framework is resilient against impersonation attacks.	The attacker may undertake a vehicle impersonation attack by intercepting the login message and obtaining the secret values from the vehicle’s smart card in an unlawful manner to listen in on, intercept, and change any message in the public communication channel.	Assume the attacker intercepts the message and attempts to construct another acceptable message that the network will validate. It is never going to happen because each and every message is recorded in the ledger over the blockchain maintained among all the RSUs and controller nodes in the network. So, any unlawful activity may not get a positive response from the rest of the network. Furthermore, guessing all of the unknown restrictions in polynomial time is impossible. As a result, the attacker will not be able to construct or guess further legitimate messages in polynomial time. Further, it is also not feasible to impersonate the vehicles by altering the message. As a legitimate message cannot be computed by the attacker unless he or she has access to all of the parameters necessary to calculate it, including the nonce of the sink node.
**P2**	The proposed framework is secured against offline password guessing attacks.	A password guessing attack is one in which an attacker attempts to impersonate a user by repeatedly guessing his password or other login information. Password guessing attacks may be carried out online by connecting to a server and trying to guess a user’s password. There are no limits on how many times an adversary may try to login in this version of the attack, unlike the offline version, which does. In the offline variant, an adversary obtains a user’s password-related data (e.g., a hashed password) and then repeatedly attempts to guess a password while comparing the hashed version to the intercepted one.	If the attacker wants to launch an offline password guessing attack on the vehicle, the attacker must first get the stored settings from the smart card. Two scenarios have been presumptively considered. One in which the attacker has stolen the vehicle’s smart card, and the other in which it is presumed that most users utilize low entropy IDs and passwords for memorizing purposes, which can be readily guessed in polynomial time. If an attacker manages to get its hands on the vehicle’s secret information, the attacker will still be unable to guess the proper password simultaneously in polynomial time. This is because no one can know a vehicle’s true identify except the vehicle itself through its smart card or messages communicated over the public channel since our approach uses blockchain to establish identity protection and passwords are safeguarded by a non-invertible one-way hash function. Hence, the attacker is unable to get access to the user’s personal information.
**P3**	Replay attacks can be protected by the proposed framework.	As the name suggests, this kind of attack involves an attacker intercepting and then fraudulently delaying or resending an already intercepted secure network communication. It is common for an attacker to re-transmit previously delivered communications in order to verify that a certain message was sent by the intended sender, hoping that this time the recipient would make a mistake and do what the attacker wants.	It is possible that a malicious actor may attempt to replay previous messages. Every time a message is sent using the proposed framework, it generates a new random number. Assume another scenario in which an antagonist has obtained one of the CRPs for a PUF and is attempting to reuse a prior challenge. This is why a CRP should never be reused. By allowing PUFs to be reprogrammed after each CRP, the system will be impervious to replay attacks. As a result, replay attacks are not a concern for the proposed methodology under development.
**P4**	The proposed framework is guarded from linking attacks.	In such kinds of attacks, to discover the real world identification of an incognito node, an adversary, who might be a controller node or cloud storage node, connects various data in the cloud or blockchain transactions with the same ID.	Each transaction in layer 3 is assigned a unique public key by the overlay nodes. Separate cloud accounts are used to verify the identity of each connected device. This makes it impossible for an attacker to connect data from various devices belonging to the same user.
**P5**	The proposed framework can protect against man-in-the-middle attacks.	An adversary node breaches the communication between two nodes in the network and obtains or compromises the information they communicate in a man-in-the-middle attack. An adversary may capture a ledger wallet by installing a malicious program on a target node and changing the address of the destination of blockchain transactions.	The information on all of the public constraints and messages’ exchange in sessions, as well as how to communicate with other roles of model, has been revealed to the adversary. Its objective is to identify all known vulnerabilities such as interception and replay the traffic, decrypt the secret keys, reveal the data of protected sessions, and threaten the legitimacy of entities. By implementing the framework, these back ends check of an attack can be detected. There are certain models that report safe, but others describe how the harm may be done and whose security objective cannot be fulfilled if the model is not secure enough. As proposed by Theorem 1, the proposed framework is protected against passive and active attacks, such as replay and man-in-the-middle attacks.Let us consider at any time a PUF gets its hands on one of the CRPs, an adversary can attempt to repeat an earlier challenge. As a result, it is preferred that a CRP be never reused. After each CRP, PUFs may be reconfigured, making the system resistant to man-in-the-middle attacks.
**P6**	Attacks such as Denial ofService(DOS) or Distributed Denial ofService(DDOS) can not affect the network security guarded with the proposed framework.	Attacks such as Distributed Denial of Service (DdoS) or Denial of Service (DOS) are meant to take advantage of weak spots in a system. This is performed by flooding a program with more requests than it can process, exceeding its network card’s capacity. Blockchain servers are overloaded with queries, which cause them to lose connectivity to other apps.	Before being sent to the global blockchain, all transactions in a local blockchain are authorized by the RSU or controller nodes. The global blockchain transactions are invalid on the local blockchain because the local blockchain and the global blockchain use different encryption algorithms. In the region cluster, a vehicle can only connect with another vehicle if the RSU has created a shared key between them. To avoid sending transactions to other vehicles in the cluster, RSUs search for matches in their key-list before sending any. Depending on the RSU’s capacity, the maximum number of transactions it may accept is limited. As soon as the limit is reached, vehicles are prevented from transmitting transactions to the destination vehicle.
**P7**	The proposed framework can defend well against the 51% attack.	An attacker must gain control of all the mining power on a specific blockchain in order to commit a 51% attack. With a mining advantage of more than 50% and the ability to mine faster than everyone else, the adversaries are doing quite well.	Based on the consensus method, the attack may be handled during the validation of neighbors or by other layer 2 nodes entirely via mutual authentication.
**P8**	Public BC modification attack is not possible in the proposed framework.	False blocks are advertised as the longest ledger by the attacker. As a result, every node acknowledges the ledger maintained by the attacker as the authentic ledger.	Layer 2 nodes, such as RSU and controllers, are limited in the number of blocks they can create in a given period of time. This restricts the number of malicious blocks that a layer 2 node may add, preventing the attacker from producing a ledger that is longer than the genuine ledger.
**P9**	Physical and cloning attacks are absolutely not possible with the proposed framework.	The investigation of information systems in order to uncover the concealed features of devices and systems by making use of the attributes of their implementation is known as cryptanalysis. One sort of cryptanalysis is known as physical attacks.	A vehicle or an IoV device might be cloned by an adversary to seem legitimate. An adversary may clone a device if it is physically compromised and the secrets from the seized device are extracted. The employment of PUFs, on the other hand, makes it exceedingly difficult for an opponent to conduct such attacks. In order to reliably assess PUF delays, a cloning attack on PUFs would require the deployment of intrusive procedures, which are not economically practical. To protect IoT devices with PUFs against physical and cloning threats, PUFs have been proven in [40,41] to be effective.
**P10**	The proposed framework is immune to side-channel attacks.	A security vulnerability known as a “side-channel attack” aims to harvest information from a chip or a system via an open channel. Various physical characteristics may be measured or analyzed to do this. Side-channel attacks are made possible by the ease with which an attacker may get access to IoT devices. Timing, power monitoring, electromagnetic attacks, and differential fault analysis are prominent examples of attacks in this category.	Statistical measurement of the time needed by a CPU to complete cryptographic operations is often used in timing attacks to discover the secret key. PUFs, on the other hand, employ a challenge response model instead of secret keys, making it more difficult to correctly measure the timing delays of a circuit in an IC. In addition, PUFs are deemed isochronous, which makes them immune to timing attacks. Attacks that rely on power consumption monitoring during calculations are known as power monitoring attacks. A data analysis method has been used by [42] to demonstrate a power side-channel attack against PUFs. They demonstrated that the number of zeros and ones stored in the latches of an arbiter PUF may be determined by utilizing power consumption information. PUFs, on the other hand, may be made safe against these attacks if the amount of zeroes and ones in the latches remains consistent. Electromagnetic attacks are a lot more difficult to carry out than power-monitoring attacks. As with power analysis attacks, the PUF may be protected against electromagnetic attacks by decreasing current fluctuations. Differential fault analysis is performed by exposing security hardware to aberrant environmental circumstances in order to introduce defects within it. Physical data corruption in cryptographic systems is often exploited by these approaches. Because of their sensitivity to temperature and voltage fluctuations, certain PUF types (such as delay-based PUFs) may be exploited by an adversary, although the physical data contained inside these PUFs cannot be exploited.

**Table 4:** Parameters Considered for Simulation.

Parameter	Year
**Speed of Vehicle**	10–60 km/h
**Number of Vehicles**	1000
**Communication Range of Vehicles**	300 m
**Communication Range of RSUs**	1000 m
**Routing Protocol**	AODV
**Simulation Time**	1000 s
**Wireless Protocol**	802.11a/802.11p
**Area**	10 km^2^

**Table 5:** Parameter Specifications of IEEE 802.11a and IEEE 802.11p.

Parameters	IEEE 802.11a	IEEE 802.11p
Data Transmission Rate	Upto 54 Mbps	Upto 27 Mbps
Channel Bandwidth	20 MHz	10 MHz
Guard Time	0.8 µs	1.6 µs
Subcarrier Spacing	0.3125 MHz	0.15625 MHz
Orthogonal Frequency Division Multiplexing Symbol Duration	4 µs	8 µs
Preamble Duration	16 µs	32 µs
Subcarrier Count	52	52
Coding Rate	Upto ¾	Upto ¾
Basic Modulations Used	Binary Phase Shit Keying (BPSK), Quadrature Phase Shift Keying (QPSK)	Binary Phase Shit Keying (BPSK), Quadrature Phase Shift Keying (QPSK)
Quadrature Amplitude Modulation (QAM)	16QAM, 64QAM	16QAM, 64QAM

**Table 6:** Security Comparison of Proposed Framework with Related Schemes.

Serial Number	Proposed Framework		[19]	[20]	[21]	[22]	[23]	[24]	[25]	[26]	[28]	[29]	[31]	[27]	[32]
	Possibility of Attack	Attack Defense Measurement													
**P1**	Least Probable	Adequate	Y	Y	Y	Y	N	Y	Y	Y	Y	N	Y	Y	Y
**P2**	Least Probable	Adequate	N	Y	N	N	N	Y	N	N	N	N	Y	N	N
**P3**	Improbable	High	Y	Y	Y	Y	Y	N	Y	Y	Y	Y	Y	N	Y
**P4**	Improbable	High	N	N	N	Y	N	N	Y	N	N	N	N	N	N
**P5**	Improbable	High	Y	N	Y	N	N	Y	Y	N	N	N	N	N	Y
**P6**	Improbable	Very High	N	N	Y	N	Y	N	N	N	N	Y	Y	Y	Y
**P7**	Improbable	Very High	N	N	Y	N	N	N	N	N	N	N	N	N	N
**P8**	Improbable	Very High	N	N	Y	N	N	N	N	N	N	N	N	N	N
**P9**	Improbable	Very High	N	N	N	N	N	N	N	N	N	N	N	N	N
**P10**	Improbable	Very High	N	N	N	N	N	N	N	N	N	N	N	N	N

Note: Y: Yes; N: No.

**Table 7:** Calculation of Computational Overhead.

Paper	Major Computation Overhead
[19]	*3B + 5vM + (3v + 1)A + (3v + 2)H + M + vE*
[20]	*20H*
[21]	*12H + 2log2vH*
[22]	*16H*
[23]	*(6M + 4A + 2H)t + (6M + 2A + S + 2H)s*
[24]	*17H*
[25]	*(n + 2) H*
[26]	*13M + 3A + (8 + 2L)H + 9SED*
[28]	*22H*
[29]	*3M(t + s)*
[31]	*12H + 6X + 2SED*
[27]	*3P + N(M + O)*
[32]	*5M + (3 + n)H+(n + 2)A*
Proposed	(16 + log_2_v)H
Notations Used in Computational Cost Calculation	
*H*: Hash Operation	*SED*: symmetric key encryption/decryption
*L*: the length of forward and backward hash chain	*E*: Encryption Operation
*n*: Number of random integers generated by RSUs	*X*: modular exponentiation
*M*: Scalar Multiplication Operation	*N*: no of signature verifications
*I*: Inversion Operation	*P*: Pairing
*A*: Addition Operation	*O*: Map to Point
*S*: Subtraction Operation	*s*: server operations
*B*: Bilinear Pairing Operation	*t*: tag operations

**Table 8:** Comparison of Computational Overhead.

Paper	Year	Cost per Vehicle (in ms)
[19]	2021	38.478
[20]	2021	2.802
[21]	2021	0.029
[22]	2021	5.12
[23]	2021	1.404
[24]	2020	0.034
[25]	2020	(n + 2) × 0.001
[26]	2019	362.5
[28]	2019	7.04
[29]	2019	6.5353604
[31]	2019	2.098
[27]	2019	50.5143
[32]	2019	4.852
Proposed	-	0.0337

**Table 9:** Comparison of Transaction Overhead.

Paper	Year	Communication Cost (in bits)
[19]	2021	2912
[20]	2021	4416
[21]	2021	1032
[22]	2021	1344
[23]	2021	1280
[24]	2020	2496
[25]	2020	832
[26]	2019	758
[28]	2019	842
[29]	2019	1440
[31]	2019	3024
[13]	2019	960
[32]	2019	1184
Proposed	**-**	3584

## Data Availability

There are no available data to be stated.
